# Modular Structure of the Weyl Algebra

**DOI:** 10.1007/s00220-022-04344-7

**Published:** 2022-03-19

**Authors:** Roberto Longo

**Affiliations:** Dipartimento di Matematica, Università di Roma Tor Vergata, Via della Ricerca Scientifica, 1, 00133 Rome, Italy

## Abstract

We study the modular Hamiltonian associated with a Gaussian state on the Weyl algebra. We obtain necessary/sufficient criteria for the local equivalence of Gaussian states, independently of the classical results by Araki and Yamagami, Van Daele, Holevo. We also present a criterion for a Bogoliubov automorphism to be weakly inner in the GNS representation. The main application of our analysis is the description of the vacuum modular Hamiltonian associated with a time-zero interval in the scalar, massive, free QFT in two spacetime dimensions, thus complementing the recent results in higher space dimensions (Longo and Morsella in The massive modular Hamiltonian. arXiv:2012.00565). In particular, we have the formula for the local entropy of a one-dimensional Klein–Gordon wave packet and Araki’s vacuum relative entropy of a coherent state on a double cone von Neumann algebra. Besides, we derive the type ${III}_1$ factor property. Incidentally, we run across certain positive selfadjoint extensions of the Laplacian, with outer boundary conditions, seemingly not considered so far.

## Introduction

The Heisenberg commutation relations are at the core of Quantum Mechanics. From the mathematical viewpoint, they have a more transparent formulation in Weyl’s exponential form. If *H* is a real linear space equipped with a non-degenerate symplectic form $\beta$, one considers the free ${}^*$-algebra *A*(*H*) linearly generated by the (unitaries) *V*(*h*), $h\in H$, that satisfy the commutation relations (CCR)

1 $$\begin{aligned} V(h+k) = e^{i\beta (h,k)}V(h)V(k), \quad h,k\in H, \end{aligned}$$

$V(h)^* = V(-h)$. The Weyl algebra *A*(*H*) admits a unique $C^*$ norm, so its $C^*$ completion is a simple $C^*$-algebra, the Weyl $C^*$-algebra $C^*(H)$. The representations, and the states, of *A*(*H*) and of $C^*(H)$ are so in one-to-one correspondence. We refer to [8, 14, 34] for the basic theory.

For a finite-dimensional *H*, von Neumann’s famous uniqueness theorem shows that all representations of $C^*(H)$, with $V(\cdot )$ weakly continuous, are quasi-equivalent. As is well known, in Quantum Field Theory (QFT) one deals with infinitely many degrees of freedom and many inequivalent representations arise, see [20].

Due to the relations (1), a state on $C^*(H)$ is determined by its value on the Weyl unitaries; a natural class of states is given by the ones with Gaussian kernel. A state $\varphi _\alpha$ is called Gaussian, or quasi-free, if

$$\begin{aligned} \varphi _\alpha \big (V(h)\big ) = e^{-\frac{1}{2} \alpha (h,h)}, \end{aligned}$$

with $\alpha$ a real bilinear form $\alpha$ on *H*, that has to be compatible with $\beta$.

Assuming now that *H* is separating with respect to $\alpha$, as is the case of a local subspace in QFT, the GNS vector associated with $\varphi _\alpha$ is cyclic and separating for the von Neumann algebra ${{\mathcal {A}}}(H)$ generated by $C^*(H)$ in the representation. So there is an associated Tomita–Takesaki modular structure, see [41], that we are going to exploit in this paper.

Modular theory is a deep, fundamental operator algebraic structure that is widely known and we refrain from explaining it here, giving for granted the reader to be at least partly familiar with that. We however point out two relevant aspects for our work. The first one is motivational and concerns the growing interest on the modular Hamiltonian in nowadays physical literature, especially in connection with entropy aspects (see e.g. refs in [28]). The other aspect concerns the crucial role taken by the modular theory of standard subspaces, see [27]; this general framework, where Operator Algebras are not immediately visible, reveals a surprisingly rich structure and is suitable for applications of various kind. Most of our paper will deal with standard subspaces.

Our motivation for this paper is the description of the local modular Hamiltonian associated with the free, massive, scalar QFT in $1+1$ spacetime dimension, in order to complement the higher dimensional results, that were obtained after decades of investigations [30]. We give our formula in Sect. 5.2. Although the present formula could be guessed from the higher dimensional one, its proof is definitely non-trivial because the deformation arguments from the massless case are not directly available now, due to the well known infrared singularities; indeed the free, massless, scalar QFT does not exist in $1+1$ dimension.

As a consequence, we compute the local entropy of a low dimensional Klein–Gordon wave packet. This gives also Araki’s vacuum relative entropy of a coherent state on a local von Neumann algebra the free, massive, scalar QFT, now also in the $1+1$ dimension case. We refer to [9, 28–30] for background results and explanation of the context. We also show the type ${III}_1$ factor property for the net of local von Neumann algebras associated with the free, massive, scalar QFT on a low dimensional Minkowski spacetime.

We now briefly describe part of the background of our work. The Canonical Commutation Relations (1) and Anti-Commutation Relations are ubiquitous and intrinsic in Quantum Physics. The study of the corresponding linear symmetries (symplectic transformations, CCR case) is a natural problem; the automorphisms of the associated operator algebras are called Bogoliubov automorphisms, see [14, 15]. The classical result of Shale [39] characterises the Bogoliubov automorphisms that are unitarily implementable on the Fock representation. Criteria of unitary implementability in a quasi-free representation were given by Araki and Yamagami [5], van Daele [42] and Holevo [23], these works are independent of the modular theory, although the last two rely on the purification construction, that originated in the classical paper by Powers and Størmer in the CAR case [36]. Woronowicz partly related the purification map to the modular theory and reconsidered the CAR case [43]. However, the modular structure of the Weyl algebra has not been fully exploited so far, although the CCR case is natural to be studied from this point of view.

We work in the context of the standard form of a von Neumann algebra studied by Araki, Connes and Haagerup [3, 11, 21]. If an automorphism of a von Neumann algebra in standard form is unitarily implementable, then it is canonically implementable; so we know where to look for a possible implementation. This will provide us with a criterion for local normality that is independent of the mentioned previous criteria, we however make use of Shale’s criterion. We shall give necessary/sufficient criteria for the quasi-equivalence of Gaussian states in terms of the modular data.

A key point in our analysis concerns the cutting projection on a standard subspace studied in [9]. On one hand, this projection is expressed in terms of the modular data, on the other hand it has a geometric description in the QFT framework. The cutting projection is thus a link between geometry and modular theory, so it gives us a powerful tool.

Among our results, we have indeed necessary/sufficient criteria for the quasi-equivalence of two Gaussian states $\varphi _{\alpha _1}$, $\varphi _{\alpha _2}$ on $C^*(H)$, in terms of the difference of certain functions of the modular Hamiltonians, that are related to the cutting projections. However, our present applications to QFT are based on our general analysis, not directly to the mentioned criteria.

The following diagram illustrates the interplay among the three equivalent structures associated with standard subspaces and the geometric way out to QFT: 
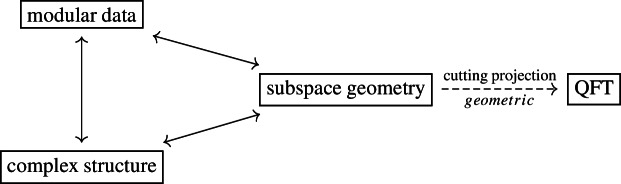


Our paper is organised as follows. We first study the modular structure of standard subspaces, especially in relations with polarisers and cutting projections. We then study the local normality/weak innerness of Bogoliubov transformations, and the quasi-equivalence of Gaussian states, in terms of modular Hamiltonians and other modular data. Finally, we present our mentioned applications in Quantum Field Theory. We also includes appendices, in particular concerning inequalities and functional calculus for real linear operators in the form we shall need. Finally, we point out certain positive selfadjoint extensions of the Laplacian, naturally arising via the inverse Helmholtz operator, that might have their own interest.

## Basic Structure

This section contains the analysis of some general, structural aspects related to closed, real linear subspaces of a complex Hilbert space, from the point of view of the modular theory.

### One-particle structure

Let *H* be a real vector space. A *symplectic form* $\beta$ on *H* is a real, bilinear, anti-symmetric form on *H*. We shall say that $\beta$ is *non-degenerate* on *H* if

$$\begin{aligned} \ker \beta \equiv \{h\in H: \beta (h,k) = 0 ,\ \forall k\in H\} = \{0\}\,. \end{aligned}$$

We shall say that $\beta$ is *totally degenerate* if $\ker \beta = H$, namely $\beta =0$. A *symplectic space* is a real linear space *H* equipped with a symplectic form $\beta$.

Given a symplectic space $(H,\beta )$, a real scalar product $\alpha$ on *H* is *compatible* with $\beta$ (or $\beta$ is compatible with $\alpha$) if the inequality

2 $$\begin{aligned} \beta (h,k)^2\le \alpha (h,h) \alpha (k,k) \ , \quad h,k\in H, \end{aligned}$$

holds. Given a compatible $\alpha$, note that $\ker \beta$ is closed (w.r.t. $\alpha$), $\beta = 0$ on $\ker \beta$ and $\beta$ is non-degenerate on $(\ker \beta )^\perp$. Clearly, $\beta$ extends to a symplectic form on the completion ${\bar{H}}$ of *H* w.r.t. $\alpha$, compatible with the extension of $\alpha$. (However $\beta$ may be degenerate on ${\bar{H}}$ even if $\beta$ is non-degenerate on *H*.)

A *one-particle structure* on *H* associated with the compatible scalar product $\alpha$ (see [24]) is a pair $({{\mathcal {H}}},\kappa )$, where $\mathcal{H}$ is a complex Hilbert space and $\kappa : H\rightarrow \mathcal{H}$ is a real linear map satisfying

- $$\Re (\kappa (h_1),\kappa (h_2))= \alpha (h_1,h_2)$$ and $$\Im (\kappa (h_1),\kappa (h_2))= \beta (h_1,h_2)$$, $$h_1,h_2\in H$$,
- $\kappa (H)+i\kappa (H)$ is dense in ${{\mathcal {H}}}$.

Note that $\kappa$ is injective because

3 $$\begin{aligned} h\in H,\ \kappa (h) = 0 \Rightarrow \Re (\kappa (h),\kappa (h)) = 0 \Rightarrow \alpha (h,h) = 0 \Rightarrow h = 0. \end{aligned}$$

With ${\bar{H}}$ the completion of ${\bar{H}}$ w.r.t. $\alpha$, $\beta$ extends to a compatible symplectic form on ${\bar{H}}$. Then $\kappa$ extends to a real linear map ${\bar{\kappa }}:{\bar{H}}\rightarrow {{\mathcal {H}}}$ with $({{\mathcal {H}}},{\bar{\kappa }})$ a one-particle structure for ${\bar{H}}$.

In the following proposition, we shall anticipate a couple of facts explained in later sections. The uniqueness can be found in [24]; the existence is inspired by [34].

#### Proposition 2.1

Let *H* be a symplectic space with a compatible scalar product $\alpha$. There exists a one-particle structure $({{\mathcal {H}}}, \kappa )$ on *H* associated with $\alpha$. It is unique, modulo unitary equivalence; namely, if $(\mathcal{H}',\kappa ')$ is another one-particle structure on *H*, there exists a unitary $U: \mathcal{H}\rightarrow \mathcal{H}'$ such that the following diagram commutes: 
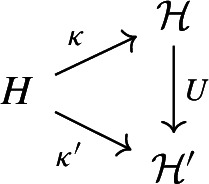


#### Proof

Uniqueness. The linear map $U: \kappa (h) \mapsto \kappa '(h)$ is well defined on $\kappa (H)$ by (3). Moreover, it extends to a complex linear map $\kappa (H)+i\kappa (H) \rightarrow \kappa '(H)+i\kappa '(H)$ and is isometric because

$$\begin{aligned}&||\kappa (h) + i \kappa (k)||^2 = ||\kappa (h)||^2 + ||\kappa (k)||^2 + 2\Re (\kappa (h), i \kappa (k)) \\&\quad = ||\kappa (h)||^2 + ||\kappa (k)||^2 - 2\Im (\kappa (h), \kappa (k)) \\&\quad = \alpha (h,h) + \alpha (k,k) -2\beta (h,k) = ||\kappa '(h) + i \kappa '(k)||^2, \end{aligned}$$

so *U* extends to a unitary operator with the desired property.

*Existence.* By replacing *H* with its completion w.r.t. $\alpha$, we may assume that *H* is complete. Suppose first that $\beta$ is totally degenerate, i.e. $\beta =0$, and let $H_{{\mathbb {C}}}$ the usual complexification of *H*, namely $H_{{\mathbb {C}}}= H \oplus H$ as real Hilbert space with complex structure given by the matrix $$i = \begin{bmatrix} 0 &{}\quad -1 \\ 1 &{}\quad 0 \end{bmatrix}$$. Then $\kappa : h\in H\mapsto h\oplus 0\in H_{{\mathbb {C}}}$ is a one-particle structure on *H* associated with $\alpha$.

Suppose now that $\beta$ is non-degenerate and consider the polariser $D_H$ (Sect. 2.2). If $\ker (D_H^2 + 1) =\{0\}$, i.e. *H* is separating (see Lemma 2.2), the orthogonal dilation provides a one-particle structure on *H* associated with $\alpha$ (Sect. 2.4). If $D^2_H = -1$, then $D_H$ is a complex structure on *H*, so the identity map is a one-particle structure. Taking the direct sum, we see that a one-particle structure exists if $\beta$ is non-degenerate.

The existence of a one-particle structure then follows in general because $H = H_a\oplus H_f$, where the restriction of $\beta$ to $H_a$ is totally degenerate and to $H_f$ is non-degenerate. $\quad \square$

### Polariser

Let $H\subset \mathcal{H}$ be a closed, real linear subspace of the complex Hilbert space ${{\mathcal {H}}}$. By the Riesz lemma, there exists a unique bounded, real linear operator $D_H$ on *H* such that

4 $$\begin{aligned} \beta (h,k) = \alpha (h, D_H k) ,\quad h,k\in H, \end{aligned}$$

with $$\alpha (\cdot ,\cdot ) = \Re (\cdot ,\cdot )$$, $$\beta (\cdot ,\cdot ) = \Im (\cdot ,\cdot )$$

We have

$$\begin{aligned} ||D_H|| \le 1, \quad D_H^* = - D_H. \end{aligned}$$

The operator $D_H$ is called the *polariser* of *H*. As

$$\begin{aligned} \Im (h, k) = -\Re (h, ik) = - \Re (h, E_H ik) ,\quad h,k\in H, \end{aligned}$$

we have one of our basic relations

5 $$\begin{aligned} D_H = - E_H i |_H, \end{aligned}$$

where $E_H$ is the orthogonal projection onto *H*.

Let $H' = (iH)^{\perp _{{\mathbb {R}}}}$ be the *symplectic complement* of *H*. We shall say that *H* is *factorial* if $H\cap H'= \{0\}$.

#### Lemma 2.2

We have

6 $$\begin{aligned} \mathrm{ker}(D_H^2 + 1) = H\cap i H, \end{aligned}$$

thus *H* is separating iff $\ker (D_H^2 + 1) = \{0\}$. Furthermore,

7 $$\begin{aligned} \mathrm{ker}(D_H) = \ker \beta = H\cap H' . \end{aligned}$$

thus *H* is factorial iff ker$(D_H) = \{0\}$.

#### Proof

As $D_H = - E_H i |_H$, with $E_H$ the orthogonal projection of ${{\mathcal {H}}}$ onto *H* (5), we have

8 $$\begin{aligned} D_H^2 = E_H i E_H i |_H = - E_H E_{iH}|_H \end{aligned}$$

so, if $$h\in H$$,

$$\begin{aligned} (D_H^2 + 1)h= 0 \Leftrightarrow E_H E_{iH} h = h \Leftrightarrow h \in H\cap iH, \end{aligned}$$

showing the first part of the lemma.

Last assertion follows as

$$\begin{aligned} \ker \beta = {{\,\mathrm{ran}\,}}(D_H)^\perp = \ker (D_H^*) = \ker (D_H) \end{aligned}$$

and clearly $$\ker \beta = H\cap H'$$. $$\quad \square $$

#### Proposition 2.3

$$h \in \ker (D_H^2 + 1) \Leftrightarrow ||D_H h|| = ||h|| \Leftrightarrow D_H h = -ih$$.

#### Proof

Let $h\in \ker (D_H^2 + 1)$, thus $D_H^2 h = - h$, so $||D^2_H h|| = ||h||$ and this implies $||D_H h|| = ||h||$ because $||D_H|| \le 1$. Thus $||E_H i h|| = ||h|| = ||ih||$, so $h\in iH$; hence $h\in H\cap i H$. So $D_H h = -E_H i h = -ih$.

Conversely, assume that $D_H h = -ih$; then $ih\in H$, so $||D_H h|| = ||E_H i h||= ||h||$. Finally, assume the equality $||D_H h|| = ||h||$ to hold. Then $||E_H i h|| = ||ih||$, so $E_H i h = ih$, hence $D_H h = -E_Hi h = -ih$, so $D_H^2 = -h$, namely $h \in \ker (D_H^2 + 1)$. $\quad \square$

### Standard subspaces

Let $\mathcal{H}$ be a complex Hilbert space and *H* a closed, real linear subspace. We say that *H* is *cyclic* if $H + i H$ is dense in $\mathcal{H}$, *separating* if $H \cap i H = \{0\}$, *standard* if it is both cyclic and separating.

Let $H\subset \mathcal{H}$ be a closed, real linear subspace of ${{\mathcal {H}}}$ and $\beta = \Im (\cdot ,\cdot )$ on *H*, where $(\cdot ,\cdot )$ is the complex scalar product on ${{\mathcal {H}}}$; then $\beta$ is a symplectic form on *H* that makes it a symplectic space. Moreover, $\alpha =\Re (\cdot ,\cdot )$ is a compatible real scalar product on *H*.

An *abstract standard subspace* is a triple $(H,\alpha ,\beta )$, where *H* is a real Hilbert space, $\alpha$ is the real scalar product on *H* and $\beta$ is a symplectic form on *H* compatible with $\alpha$, so that *H* separating, that is $\ker (D^2_H + 1) = \{0\}$, with $D_H$ the polariser of *H*, see Lemma 2.2.

By Proposition 2.1, an abstract standard subspace can be uniquely identified, up to unitary equivalence, with a standard subspace of a complex Hilbert space as above.

We shall say that the abstract standard subspace $(H,\alpha ,\beta )$ is *factorial* if $\ker (D_H) = \{0\}$, namely $\beta$ is non-degenerate.

In view of the above explanations, we shall often directly deal with standard subspaces of a complex Hilbert space ${{\mathcal {H}}}$.

Given a standard subspace *H* of ${{\mathcal {H}}}$, we shall denote by $J_H$ and $\Delta _H$ the *modular conjugation* and the *modular operator* of *H*; they are defined by the polar decomposition $S_H = J_H \Delta _H^{1/2}$ of the closed, densely defined, anti-linear involution on ${{\mathcal {H}}}$

$$\begin{aligned} S_H : h + ik \mapsto h - ik, \quad h,k\in H. \end{aligned}$$

$\Delta _H$ is a non-singular, positive selfadjoint operator, $J_H$ is an anti-unitary involution and we have

9 $$\begin{aligned} J_H\Delta _H J_H = \Delta _H^{-1}. \end{aligned}$$

The fundamental relations are

$$\begin{aligned} \Delta _H^{is} H = H, \quad J_H H = H', \quad s\in {{\mathbb {R}}}, \end{aligned}$$

see [25, 27, 37]. We denote by

$$\begin{aligned} L_H = \log \Delta _H \end{aligned}$$

the *modular Hamiltonian* of *H*. We often simplify the notation setting $$ L = L_H$$ and similarly for other operators.

Assume now *H* to be standard and factorial. Let $E_H$ be the real *orthogonal projection* from ${{\mathcal {H}}}$ onto *H* as above and $P_H$ the *cutting projection*

10 $$\begin{aligned} P _H: h + h' \mapsto h, \quad h\in H,\, h'\in H'. \end{aligned}$$

$P_H:D(P_H)\subset {{\mathcal {H}}}\rightarrow {{\mathcal {H}}}$ is a closed, densely defined, real linear operator with domain $D(P_H) = H + H'$.

Recall two formulas respectively in [17] and in [9]:

11 $$\begin{aligned}&E_H = (1 + \Delta _H)^{-1} + J_H\Delta _H^{1/2} (1 + \Delta _H)^{-1} , \end{aligned}$$

12 $$\begin{aligned}&P_H = (1 - \Delta _H)^{-1} + J_H\Delta _H^{1/2} (1 - \Delta _H)^{-1} ; \end{aligned}$$

more precisely, $P_H$ is the closure of the right hand side of (12).

These formulas can be written as

13 $$\begin{aligned}&E_H = (1 + S_H )(1 + \Delta _H)^{-1} , \end{aligned}$$

14 $$\begin{aligned}&P_H = (1 + S_H ) (1 - \Delta _H)^{-1} , \end{aligned}$$

so give

15 $$\begin{aligned} P_H = E_H (1 + \Delta _H) (1 - \Delta _H)^{-1} = - E_H \coth (L_H/2). \end{aligned}$$

In the following, if $T:D(T)\subset {{\mathcal {H}}}\rightarrow {{\mathcal {H}}}$ is a real linear operator, $T|_H$ is the restriction of *T* to $D(T|_H)\equiv D(T)\cap H$, that we may consider also as operator $H\rightarrow H$ if ${{\,\mathrm{ran}\,}}(T|_H)\subset H$, as it will be clear from the context.

#### Proposition 2.4

Let $H\subset {{\mathcal {H}}}$ be a factorial standard subspace. The polariser $D_H$ of *H* and its inverse $D_H^{-1}$ are given by

16 $$\begin{aligned} D_H= & {} - E_H i|_H = i( \Delta _H -1)(\Delta _H +1)^{-1}|_H= i\tanh (L_H/2)|_H, \end{aligned}$$

17 $$\begin{aligned} D_H^{-1}= & {} P_H i|_H = - i( \Delta _H +1)(\Delta _H -1)^{-1}|_H = - i\coth (L_H/2)|_H . \end{aligned}$$

As a consequence, $P_H i|_H$ is a skew-selfadjoint real linear operator on *H*.

#### Proof

As $J_H\Delta _H J_H = \Delta _H^{-1}$, Eq. (11) gives

$$\begin{aligned} E_H = (1 + \Delta _H)^{-1} +\Delta _H(1 + \Delta _H)^{-1} J \Delta _H^{1/2}, \end{aligned}$$

therefore

18 $$\begin{aligned} E_H ih= & {} \Big ( (1 + \Delta _H)^{-1} +\Delta _H(1 + \Delta _H)^{-1} S_H \Big ) ih = (1 + \Delta _H)^{-1}ih - \Delta _H(1 + \Delta _H)^{-1} ih\nonumber \\= & {} (1 - \Delta _H)(1 + \Delta _H)^{-1}ih , \end{aligned}$$

$$h\in H$$, thus

19 $$\begin{aligned} E_H i |_{H} = (1 - \Delta _H)(1 + \Delta _H)^{-1}i|_H. \end{aligned}$$

As $D_H = - E_H i |_H$ (5), Eq. (16) is proved.

Concerning formula (17), since *H* is left invariant by $(\Delta _H + 1)(\Delta _H -1)^{-1}i$, from (15) we get

$$\begin{aligned} P_H i|_H = - E_H \coth (L_H/2)i|_H = - i \coth (L_H/2)|_H = - i(\Delta _H + 1)(\Delta _H -1)^{-1}|_H . \end{aligned}$$

So $P_H i|_H$ is skew-selfadjoint because *H* is globally $\Delta _H^{is}$-invariant, $s\in {{\mathbb {R}}}$ [30, Prop. 2.2]. $\quad \square$

#### Corollary 2.5

We have

20 $$\begin{aligned} \sqrt{1 + D_H^{2}}= & {} 2(\Delta _H^{1/2} + \Delta _H^{-1/2})^{-1}|_H = \frac{1}{\cosh (L_H/2)}\Big |_H. \end{aligned}$$

21 $$\begin{aligned} D_H^{-1}\sqrt{1 + D_H^{2}}= & {} - 2i(\Delta _H^{1/2} - \Delta _H^{-1/2})^{-1}|_H = - i \frac{1}{\sinh (L_H/2)}\Big |_H; \end{aligned}$$

#### Proof

By Proposition 2.4$D_H = i\tanh (L_H/2)|_H$, thus

22 $$\begin{aligned} D_H^2 = -\tanh ^2(L_H/2)|_H, \end{aligned}$$

so $D_H^2$ is a bounded selfadjoint operator on *H* (as real linear operator). Therefore

23 $$\begin{aligned} 1 + D_H^2 = \big (1 -\tanh ^2(L_H/2)|_H\big )\big |_H = \frac{1}{\cosh ^2(L_H/2)}\Big |_H , \end{aligned}$$

thus (20) holds.

By Proposition 2.4 we then have

$$\begin{aligned} D_H^{-1}\sqrt{1 + D_H^{2}} = - i \frac{\coth (L_H/2) }{\cosh (L_H/2)}\Big |_H = - i \frac{1}{\sinh (L_H/2)}\Big |_H. \end{aligned}$$

$$\quad \square $$

The following corollary follows at once from [31]. The type of a subspace refers to the second quantisation von Neumann algebra.

#### Corollary 2.6

We have

24 $$\begin{aligned} E_H E_{H'}|_H = 1 + D_H^{2} . \end{aligned}$$

Therefore, *H* is a type *I* subspace iff $1 + D_H^{2}$ is a trace class operator.

#### Proof

By [31, Lemma 2.4], we have $E_H E_{H'}|_H = 4\Delta _H(1 + \Delta _H)^{-2}|_H$; by (23), we have

$$\begin{aligned} 4\Delta _H(1 + \Delta _H)^{-2}|_H = \frac{1}{\cosh ^2(L_H/2)}\Big |_H = 1 + D_H^{2}. \end{aligned}$$

The corollary thus follows by [31, Cor. 2.6]. $\quad \square$

By (24) and (8), we have the nice identity

25 $$\begin{aligned} E_H E_{H'}|_H + E_H E_{iH}|_H = 1 . \end{aligned}$$

Let $(H,\alpha _k, \beta )$ be abstract standard subspaces, $k=1,2$, and suppose that $\alpha _1$ is equivalent to $\alpha _2$, thus there exists a bounded, positive linear map $T: H\rightarrow H$ with bounded inverse such that $\alpha _2(h,k) = \alpha _1(h,Tk)$. Then

$$\begin{aligned} \alpha _1(h, D_1 k) = \beta (h,k) = \alpha _2(h, D_2 k) = \alpha _1(h, T D_2 k), \end{aligned}$$

thus $$D_1 = T D_2$$.

### Orthogonal dilation

Let *H* be a real Hilbert space, with real scalar product $\alpha$, and consider the doubling

$$\begin{aligned} {\widetilde{H}} = H \oplus H \end{aligned}$$

(direct sum of real Hilbert spaces). We consider a symplectic form $\beta$ on *H*, that we assume to be non-degenerate and compatible with $\alpha$. Let *D* be the polariser of $\beta$ on *H* given by (4). So $\ker (D) = \{0\}$. We also assume that $\ker (1 + D^2) =\{0\}$, namely $(H,\alpha , \beta )$ is a factorial abstract subspace (6). Set

26 $$\begin{aligned} \iota = \begin{bmatrix} D &{}\quad V\sqrt{1 + D^{2}}\\ V\sqrt{1 + D^{2}} &{}\quad - D\end{bmatrix} , \end{aligned}$$

with *V* the phase of *D* in the polar decomposition, $D = V|D|$; note that *V* commutes with *D*, because *D* is skew-selfadjoint, and $V^2 = -1$ (see [7, 34]). Then $\iota$ is a unitary on ${\widetilde{H}}$ and $\iota ^2 = -1$, namely $\iota$ is a complex structure on ${\widetilde{H}}$.

Let $\mathcal{H}$ be the complex Hilbert space given by ${\widetilde{H}}$ and $\iota$. The scalar product of ${{\mathcal {H}}}$ is given by

$$\begin{aligned} (h_1 \oplus h_2, k_1 \oplus k_2) = {\widetilde{\alpha }}(h_1 \oplus h_2, k_1 \oplus k_2) + i {\widetilde{\beta }}(h_1 \oplus h_2, k_1 \oplus k_2) \end{aligned}$$

with $${\widetilde{\alpha }} \equiv \alpha \oplus \alpha $$ and $${\widetilde{\beta }}(h_1 \oplus h_2, k_1 \oplus k_2) = {\widetilde{\alpha }}(h_1 \oplus h_2, \iota (k_1 \oplus k_2))$$.

The embedding $$\kappa : H \rightarrow \mathcal{H}$$

$$\begin{aligned} \kappa : h \mapsto \kappa (h) \equiv h\oplus 0 \end{aligned}$$

satisfies the condition *b*) in Sect. 2.1, that is ${\widetilde{\alpha }}(\kappa (h),\kappa (k)) = \alpha (h,k)$ and

$$\begin{aligned} {\widetilde{\beta }}(\kappa (h),\kappa (k))= & {} {\widetilde{\alpha }}(h \oplus 0, \iota (k \oplus 0)) = {\widetilde{\alpha }}(h \oplus 0, Dk \oplus V\sqrt{1 + D^{2}}\, k))\\= & {} \alpha (h, D k) = \beta (h,k), \end{aligned}$$

$$h,k\in H$$.

#### Lemma 2.7

$\kappa (H)$ cyclic and separating in ${\widetilde{H}}$, so $\kappa$ is a one-particle structure for *H* with respect to $\alpha$ and $\kappa (H)$ is a factorial subspace.

#### Proof

$\kappa (H)$ cyclic means that the linear span of $H\oplus 0$ and $\{\iota (h\oplus 0): h\in H\}$ is dense in $\mathcal{H}$. As

$$\begin{aligned} \iota (h\oplus 0) = Dh\oplus - V\sqrt{1 + D^{2}}\, h, \end{aligned}$$

$\kappa (H)$ is cyclic iff ${{\,\mathrm{ran}\,}}(V\sqrt{1 + D^{2}})$ is dense, thus iff $\mathrm{ker}(1 + D^2) = \{0\}$. The proof is then complete by Lemma 2.2. $\quad \square$

By the above discussion $H\subset {{\mathcal {H}}}$ is a factorial standard subspace. We call $H\subset {{\mathcal {H}}}$ the *orthogonal dilation* of $(H,\beta )$ with respect to $\alpha$.

### Symplectic dilation

Let $(H,\alpha , \beta )$ be an abstract factorial standard subspace. Consider the doubled symplectic space $(H\oplus H ,{\hat{\beta }})$, where ${\hat{\beta }} = \beta \oplus -\beta$.

With *D* the polariser of $\alpha$, let $H_0 = {{\,\mathrm{ran}\,}}(D)$ and set

27 $$\begin{aligned} \iota = \begin{bmatrix} D^{-1} &{}\quad D^{-1}\sqrt{1 + D^2}\\ - D^{-1}\sqrt{1 + D^{2}} &{}\quad - D^{-1}\end{bmatrix} , \end{aligned}$$

where the matrix entries are defined as real linear operators $(H,\alpha ) \rightarrow (H,\alpha )$ with domain $H_0$. Then

$$\begin{aligned} \iota ^2 = -1 \end{aligned}$$

on $H_0 \oplus H_0$. A direct calculation shows that

28 $$\begin{aligned} {\hat{\beta }}(\iota \xi ,\iota \eta ) \equiv {\hat{\beta }}( \xi , \eta )\, ,\quad \xi ,\eta \in H_0\oplus H_0; \end{aligned}$$

setting

29 $$\begin{aligned} {\hat{\alpha }}(\xi ,\eta ) \equiv {\hat{\beta }}(\xi , \iota \eta ),\quad \xi ,\eta \in H_0\oplus H_0, \end{aligned}$$

we have a real scalar product ${\hat{\alpha }}$ on $H_0\oplus H_0$ which is compatible with ${\hat{\beta }}$. Let ${\hat{{{\mathcal {H}}}}}$ be the completion of $H_0\oplus H_0$ with respect to ${\hat{\alpha }}$; then ${\hat{{{\mathcal {H}}}}}$ is a real Hilbert space with scalar product still denoted by ${\hat{\alpha }}$.

By (28), (29), $\iota$ preserves ${\hat{\alpha }}$, so the closure of $\iota$ is a complex structure on *H*, and $\iota$ is the polariser of ${\hat{\alpha }}$ w.r.t. ${\hat{\beta }}$. Then ${\hat{\beta }}$ extends to a symplectic form on ${{\mathcal {H}}}$ compatible with ${\hat{\alpha }}$. So ${\hat{{{\mathcal {H}}}}}$ is indeed a complex Hilbert space and $H\subset {\hat{{{\mathcal {H}}}}}$ is a real linear subspace, where *H* is identified with $H\oplus 0$.

We call $H\subset {\hat{{{\mathcal {H}}}}}$ the *symplectic dilation* of $(H,\beta )$ with respect to $\alpha$.

#### Proposition 2.8

*H* is a factorial standard subspace of the symplectic dilation ${\hat{{{\mathcal {H}}}}}$. Therefore the symplectic and the orthogonal dilations are unitarily equivalent.

#### Proof

*H* is complete, thus closed in ${\hat{{{\mathcal {H}}}}}$. Since the polariser of *H* in ${\hat{{{\mathcal {H}}}}}$ is equal to *D*, the proposition follows by Lemma 2.2. $\quad \square$

## Bogoliubov Automorphisms

In this section we study symplectic maps that promote to unitarily implementable automorphisms on the Fock space.

Given a symplectic space $(H, \beta )$, we consider the *Weyl algebra* *A*(*H*) associated with *H*, namely the free ${}^*$-algebra complex linearly generated by the Weyl unitaries *V*(*h*), $h\in H$, that satisfy the commutation relations

$$\begin{aligned} V(h+k) = e^{i\beta (h,k)}V(h)V(k), \quad V(h)^* = V(-h), \quad h,k\in H. \end{aligned}$$

The $C^*$ envelop of *A*(*H*) is the *Weyl* $C^*$-*algebra* $C^*(H)$. If $\beta$ non-degenerate, there exists a unique $C^*$ norm on *A*(*H*) and $C^*(H)$ is a simple $C^*$-algebra.

Let ${{\mathcal {H}}}$ be a complex Hilbert space and $e^{{\mathcal {H}}}$ be the Bosonic Fock Hilbert space over ${{\mathcal {H}}}$. Then we have the *Fock representation* of $C^*({{\mathcal {H}}}_{{\mathbb {R}}})$ on $e^{{\mathcal {H}}}$, where ${{\mathcal {H}}}_{{\mathbb {R}}}$ is ${{\mathcal {H}}}$ as a real linear space, equipped with the symplectic form $\beta \equiv \Im (\cdot ,\cdot )$. In the Fock representation, the Weyl unitaries are determined by their action on the vacuum vector $e^0$

30 $$\begin{aligned} V(h)e^{0} = e^{-\frac{1}{2} (h,h)}e^{h},\ h\in {{\mathcal {H}}}, \end{aligned}$$

where $e^h$ is the coherent vector associated with *h*. So the *Fock vacuum state* $\varphi = (e^0,\cdot \, e^0)$ of $C^*({{\mathcal {H}}}_{{\mathbb {R}}})$ is given by

31 $$\begin{aligned} \varphi \big (V(h)\big )= e^{-\frac{1}{2} ||h||^2},\quad h\in {{\mathcal {H}}}. \end{aligned}$$

With *H* any real linear subspace of ${{\mathcal {H}}}$, the Fock representation determines a representation of $C^*(H)$ on $e^{{\mathcal {H}}}$, which is cyclic on $e^{{\mathcal {H}}}$ iff *H* is a cyclic subspace of ${{\mathcal {H}}}$. We denote by ${{\mathcal {A}}}(H)$ the von Neumann algebra on $e^{{\mathcal {H}}}$ generated by the image of $C^*(H)$ in this representation. We refer to [8, 26, 27, 32] for details.

### Global automorphisms

Let ${{\mathcal {H}}}$ be a complex Hilbert space and $e^{{\mathcal {H}}}$ the Fock space as above. A *symplectic map* $T: D(T)\subset {{\mathcal {H}}}\rightarrow {{\mathcal {H}}}$ is a real linear map with *D*(*T*) and ran(*T*) dense, that preserves the imaginary part of the scalar product, thus $\Im (T\xi , T\eta ) = \Im (\xi ,\eta )$, $\xi ,\eta \in D(T)$.

Let $T: D(T)\subset {{\mathcal {H}}}\rightarrow {{\mathcal {H}}}$ be a symplectic map. Then

$$\begin{aligned} \Re ( iT\xi , T\eta ) = \Re (i\xi , \eta ), \quad \xi ,\eta \in D(T), \end{aligned}$$

thus $iT\xi \in D(T^*)$ and $T^*iT\xi = i\xi$ for all $\xi \in D(T)$, namely

32 $$\begin{aligned} T^*iT = i|_{D(T)} , \end{aligned}$$

therefore ker$(T) = \{0\}$, *T* is closable because $T^*$ is densely defined, and $T^{-1} = -iT^*i|_{{{\,\mathrm{ran}\,}}(T)}$, so $T^*|_{i{{\,\mathrm{ran}\,}}(T)}$ is a symplectic map too. It also follows that

33 $$\begin{aligned} T \ \text {bounded} \Longleftrightarrow T^* \ \text {bounded} \Longleftrightarrow T^{-1} \ \text {bounded} . \end{aligned}$$

We then have the associated *Bogoliubov homomorphism* $\vartheta _T$ of the Weyl algebra $A\big (D(T)\big )$ onto $A\big (\!{{\,\mathrm{ran}\,}}(T)\big )$:

$$\begin{aligned} \vartheta _T : V(\xi ) \mapsto V(T\xi ), \ \xi \in D(T). \end{aligned}$$

Let $T:{{\mathcal {H}}}\rightarrow {{\mathcal {H}}}$ be a bounded, everywhere defined symplectic map; the criterion of Shale [39] gives a necessary and sufficient condition in order that $\vartheta _T$ be unitary implementable on $e^{{\mathcal {H}}}$, under the assumption that *T* has a bounded inverse:

34 $$\begin{aligned} \vartheta _T\ \text {unitary implementable}\ \Longleftrightarrow \ T^*T - 1 \in \mathcal{L}^2({{\mathcal {H}}})\ \Longleftrightarrow \ [T,i] \in \mathcal{L}^2({{\mathcal {H}}}), \end{aligned}$$

where $[T,i] = Ti -iT = Ti(1-T^*T)$ is the commutator and $\mathcal{L}^2({{\mathcal {H}}})$ are the real linear, Hilbert–Schmidt operator on ${{\mathcal {H}}}$.

Due to the equivalence (33), the assumption $T^{-1}$ bounded in (34) can be dropped (as we assume that ran(*T*) is dense).

We shall deal with symplectic maps that, a priori, are not everywhere defined. However the following holds.

#### Lemma 3.1

Let $T: D(T)\subset {{\mathcal {H}}}\rightarrow {{\mathcal {H}}}$ be a symplectic map. Then $\vartheta _T$ is unitarily implementable iff $\vartheta _{{\overline{T}}}$ is unitarily implementable, where ${\overline{T}}$ is the closure of *T*. In this case, *T* is bounded.

#### Proof

First we show that, if $\vartheta _T$ is implemented by a unitary *U* on $e^{{\mathcal {H}}}$, then *T* is bounded. Indeed, if $\xi _n\in D(T)$ is a sequence of vectors with $\xi _n\rightarrow 0$, then $V(\xi _n) \rightarrow 1$ strongly, thus $V(T\xi _n) =UV(\xi _n)U^* \rightarrow 1$, so

$$\begin{aligned} \varphi \big ((V(T\xi _n)\big ) = e^{-\frac{1}{2} ||T \xi _n||^2} \rightarrow 1, \end{aligned}$$

with $\varphi$ the Fock vacuum state, therefore $||T \xi _n||\rightarrow 0$ and *T* is bounded.

If $\vartheta _{{\overline{T}}}$ is implemented, then $\vartheta _{T}$ is obviously implementable by the same unitary. Conversely, assume that $\vartheta _T$ is implementable by a unitary *U* on ${{\mathcal {H}}}$. So *T* is bounded. Hence ${\overline{T}}$ is a bounded, everywhere defined symplectic map. Let $\xi \in {{\mathcal {H}}}$ and choose a sequence of elements $\xi _n\in D(T)$ such that $\xi _n \rightarrow \xi$. Then

$$\begin{aligned} \vartheta _{{\overline{T}}}\big (V(\xi )\big ) = V({{\overline{T}}}\xi ) = \lim _n V(T \xi _n) = \lim _n UV(\xi _n)U^* = UV(\xi )U^*, \end{aligned}$$

so $\vartheta _{{\overline{T}}}$ is implemented by *U*. $\quad \square$

### Hilbert–Schmidt perturbations

Motivated by Shale’s criterion, we study here Hilbert–Schmidt conditions related to the symplectic dilation of a symplectic map.

We use the following *notations:* If ${{\mathcal {H}}}$ is a complex Hilbert space, $\mathcal{L}^p({{\mathcal {H}}})$ denotes the space of real linear, densely defined operators *T* on ${{\mathcal {H}}}$ that are bounded and the closure ${\bar{T}}$ belongs to the Schatten *p*-ideal with respect to the real part of the scalar product, $1\le p<\infty$. If ${{\mathcal {H}}}_1,{{\mathcal {H}}}_2$ are complex Hilbert spaces, $T\in \mathcal{L}^p({{\mathcal {H}}}_1,{{\mathcal {H}}}_2)$ means $T^* T\in \mathcal{L}^{\frac{p}{2}}({{\mathcal {H}}}_1)$. If $H\subset {{\mathcal {H}}}$ is a standard subspace, $T\in \mathcal{L}^p(H)$ means that *T* is a real linear, everywhere defined operator on *H* in the Schatten *p*-ideal with respect to the real part of the scalar product. Similarly, $T\in \mathcal{L}^p(H_1,H_2)$ means $T\in \mathcal{L}^{\frac{p}{2}}(H)$.

Let now $H\subset {{\mathcal {H}}}$ be a factorial standard subspace of the Hilbert space ${{\mathcal {H}}}$ and $C: H + H' \rightarrow H + H'$ a real linear operator. As $H + H'$ is the linear direct sum of *H* and $H'$, we may write *C* as a matrix of operators

35 $$\begin{aligned} C = \begin{bmatrix} C_{11} &{}\quad C_{12}\\ C_{21} &{}\quad C_{22} \end{bmatrix} \end{aligned}$$

(the *symplectic matrix decomposition*). Thus

$$\begin{aligned} C_{11} = P_H C |_H, \ \ C_{12} = P_H C |_{H'}, \ldots \end{aligned}$$

and $C_{11}$ is an operator $H\rightarrow H$, $C_{12}$ is an operator $H'\rightarrow H$, etc.

We want to study the Hilbert–Schmidt condition for *C*. Note that

$$\begin{aligned} C\in \mathcal{L}^2({{\mathcal {H}}}) \Longleftrightarrow E_H C E_H \in \mathcal{L}^2({{\mathcal {H}}}),\ \ E_H C E_{H^\perp } \in \mathcal{L}^2({{\mathcal {H}}})\ldots \end{aligned}$$

With $D= D_H$ the polariser and $J = J_H$ the modular conjugation, the symplectic matrix decomposition of the complex structure is

36 $$\begin{aligned} i = \begin{bmatrix} D^{-1} &{}\quad D^{-1}\sqrt{1 + D^{2}}J \\ - J D^{-1}\sqrt{1 + D^{2}} &{}\quad - J D^{-1} J \end{bmatrix}, \end{aligned}$$

as follows from (27) and the uniqueness of the dilation. Note, in particular, the identity

37 $$\begin{aligned} P_{H'}i|_H = - J D^{-1}\sqrt{1 + D^{2}} . \end{aligned}$$

#### Lemma 3.2

The following symplectic matrix representations hold:

$$\begin{aligned} E_H = \begin{bmatrix} 1 &{}\quad \sqrt{1 + D^{2}}\,J \\ 0 &{}\quad 0 \end{bmatrix},\quad E_{H^\perp } = \begin{bmatrix} 0 &{}\quad -\sqrt{1 + D^{2}}J \\ 0 &{}\quad 1 \end{bmatrix},\quad E_{H'} = \begin{bmatrix} 0 &{}\quad 0 \\ J \sqrt{1 + D^{2}} &{}\quad 1 \end{bmatrix}. \end{aligned}$$

#### Proof

We have

38 $$\begin{aligned} E_H i = \begin{bmatrix} - D &{}\quad 0\\ 0 &{}\quad 0 \end{bmatrix} \end{aligned}$$

because $$E_H i$$ is equal to $$-D$$ on *H* and zero on $$H' = iH^\perp $$. As $$E_H = -(E_H i)i $$, the first equality in the lemma follows by matrix multiplication with (36). The second equality is then simply obtained as

$$\begin{aligned} E_{H^\perp } = 1 - E_H = \begin{bmatrix} 0 &{}\quad -\sqrt{1 + D^{2}}J \\ 0 &{}\quad 1 \end{bmatrix} . \end{aligned}$$

Last equality follows as

$$\begin{aligned} E_{H'} = J E_H J \end{aligned}$$

and the symplectic matrix decomposition of *J* is $$\begin{bmatrix} 0 &{}\quad J \\ J &{}\quad 0 \end{bmatrix}$$. $\quad \square$

#### Lemma 3.3

Let $C: H + H' \rightarrow H+ H'$ be a real linear map such that $i Ci = C$, with symplectic matrix decomposition (35). We have

39 $$\begin{aligned} E_H C |_H= & {} C_{11} + \sqrt{1 + D^{2}}\, J C_{21}, \end{aligned}$$

40 $$\begin{aligned} E_{H} C i|_{H'}= & {} D C_{12}, \end{aligned}$$

41 $$\begin{aligned} E_{H'}i C |_H= & {} J D J C_{21}, \end{aligned}$$

42 $$\begin{aligned} E_{H'} C |_{H'}= & {} J \sqrt{1 + D^{2}}\, C_{12} + C_{22}. \end{aligned}$$

#### Proof

We have

43 $$\begin{aligned} E_H C = \begin{bmatrix} C_{11} + \sqrt{1 + D^{2}}\,J C_{21} &{}\quad C_{12} + \sqrt{1 + D^{2}}\, J C_{22} \\ 0 &{}\quad 0 \end{bmatrix} , \end{aligned}$$

thus

$$\begin{aligned} E_H C |_H = C_{11} + \sqrt{1 + D^{2}}\, J C_{21}\ , \end{aligned}$$

namely, (39) holds.

Since $Ci = -i C$, we have

$$\begin{aligned} E_{H} C i = - E_{H} i C = \begin{bmatrix} D &{}\quad 0 \\ 0 &{}\quad 0 \end{bmatrix}C , \end{aligned}$$

so

$$\begin{aligned} E_{H} C i = \begin{bmatrix} D C_{11} &{}\quad D C_{12} \\ 0 &{}\quad 0 \end{bmatrix} , \end{aligned}$$

thus

$$\begin{aligned} E_{H} C i|_{H'} =D C_{12} \end{aligned}$$

and (40) holds.

With $C^j = J C J$, we then get

$$\begin{aligned} E_{H'}i C |_H= & {} J E_H J iC |_H = - J E_H J C i |_H = - J E_H C^j J i|_H = J E_H C^j i J |_H \\= & {} J (E_H C^j i ) |_{H'} J = J D C^j_{12} J = J D J J C^j_{12} J = J D J C_{21} , \end{aligned}$$

so (41) holds.

Similarly, from (39) we get (42). $\quad \square$

With *H* a standard subspace, *a symplectic map of the standard subspace* *H* is a real linear map $T: H\rightarrow H$ such that

$$\begin{aligned} \Im (Th,Tk) = \Im (h,k),\quad h,k\in H, \end{aligned}$$

equivalently

$$\begin{aligned} \Re (Th,D Tk) = \Re (h, Dk),\quad h,k\in H, \end{aligned}$$

so

$$\begin{aligned} T\ \text {symplectic} \Leftrightarrow T^* D T = D; \end{aligned}$$

if *T* is invertible, we shall say that *T* is a *symplectic bijection of* *H*.

Now, let *H* be a factorial standard subspace and $T: H\rightarrow H$ a symplectic bijection. Denote by ${\widetilde{T}}$ the symplectic map $T \oplus J T J: H + H'\rightarrow H + H'$, namely ${\widetilde{T}} = TP_H + JTJ P_{H'}$, i.e.

$$\begin{aligned} {\widetilde{T}} = \begin{bmatrix} T &{} \quad 0\\ 0&{}\quad J T J \end{bmatrix} \end{aligned}$$

in the symplectic matrix description. We have

$$\begin{aligned} {\widetilde{T}} i= & {} \begin{bmatrix} T D^{-1}&{}\quad T D^{-1}\sqrt{1 + D^{2}}\, J\\ - J T D^{-1}\sqrt{1 + D^{2}}&{}\quad - J T D^{-1}J \end{bmatrix},\\ i{\widetilde{T}}= & {} \begin{bmatrix} D^{-1}T&{}\quad D^{-1}\sqrt{1 + D^{2}}\, T J\\ - J D^{-1}\sqrt{1 + D^{2}}\, T&{}\quad - J D^{-1} T J\end{bmatrix},\\ {[}{\widetilde{T}},i]= & {} \begin{bmatrix} [T,D^{-1}]&{}\quad \big [T, D^{-1}\sqrt{1 + D^{2}}\big ] J\\ - J \big [T, D^{-1}\sqrt{1 + D^{2}}\big ]&{}\quad - J [T,D^{-1}] J \end{bmatrix}. \end{aligned}$$

Note that

$$\begin{aligned} i[{\widetilde{T}},i]i = i({\widetilde{T}} i - i {\widetilde{T}})i = - i {\widetilde{T}} + {\widetilde{T}} i = [{\widetilde{T}},i]. \end{aligned}$$

#### Corollary 3.4

We have

44 $$\begin{aligned} E_H [ {\widetilde{T}},i ] |_H= & {} [T,D^{-1}] - \sqrt{1 + D^{2}}\,\big [T, D^{-1}\sqrt{1 + D^{2}}\big ], \end{aligned}$$

45 $$\begin{aligned} E_{H} \big [ {\widetilde{T}},i \big ] i|_{H'}= & {} D\big [T, D^{-1}\sqrt{1 + D^{2}}\big ] J , \end{aligned}$$

46 $$\begin{aligned} E_{H'}i \big [ {\widetilde{T}},i \big ] |_H= & {} -J D\big [T, D^{-1}\sqrt{1 + D^{2}}\big ], \end{aligned}$$

47 $$\begin{aligned} E_{H'} \big [ {\widetilde{T}},i \big ] |_{H'}= & {} J \big (\sqrt{1 + D^{2}}\,\big [T, D^{-1}\sqrt{1 + D^{2}}\big ] - [T,D^{-1}]\big ) J. \end{aligned}$$

#### Proof

We apply Lemma 3.3 with $C = \big [ {\widetilde{T}},i\big ]$. By (39), we get (44). By (40), we get (45). By (41), we get (46). By (42), we get (47). $\quad \square$

#### Proposition 3.5

$[{\widetilde{T}}, i]\in \mathcal{L}^2({{\mathcal {H}}})$ iff both the following conditions hold:

$$\begin{aligned}&\mathrm{(a)}\ [T,D^{-1}] - \sqrt{1 + D^{2}}\,\big [T, D^{-1}\sqrt{1 + D^{2}}\big ]\in \mathcal{L}^2(H),\\&\mathrm{(b)}\ D\big [T, D^{-1}\sqrt{1 + D^{2}}\big ]\in \mathcal{L}^2(H). \end{aligned}$$

#### Proof

Assume $[{\widetilde{T}}, i]\in \mathcal{L}^2({{\mathcal {H}}})$. Then the operators (44), (45) are Hilbert–Schmidt, and this implies that the operators in the statement are in $\mathcal{L}^2(H)$.

Conversely, assume that the operators in the statement are in $\mathcal{L}^2(H)$. Then the operators in Lemma 3.4 are in $\mathcal{L}^2(H)$.

Now,

$$\begin{aligned} E_{H^\perp } C E_{H^\perp } = i E_{H'}i C i E_{H'}i = - i E_{H'} C E_{H'}i , \end{aligned}$$

thus

$$\begin{aligned} E_{H^\perp } C |_{H^\perp } \in \mathcal{L}^2(H^\perp ) \Longleftrightarrow E_{H'} C |_{H'} \in \mathcal{L}^2(H'); \end{aligned}$$

moreover,

48 $$\begin{aligned} E_{H} C |_{H^\perp } \in \mathcal{L}^2(H^\perp ,H) \Longleftrightarrow E_{H} C i|_{H'} \in \mathcal{L}^2(H', H). \end{aligned}$$

We conclude that all the four matrix elements in the orthogonal decomposition of $\big [{\widetilde{T}}, i\big ]$ are in $\mathcal{L}^2({{\mathcal {H}}})$, thus $[{\widetilde{T}}, i]\in \mathcal{L}^2({{\mathcal {H}}})$. $\quad \square$

#### Corollary 3.6

Assume $[T, D^{-1}]\in \mathcal{L}^2(H)$ and $\big [T, D^{-1}\sqrt{1 + D^{2}}\big ] \in \mathcal{L}^2(H)$. Then $[{\widetilde{T}}, i]\in \mathcal{L}^2({{\mathcal {H}}})$.

#### Proof

If the assumptions are satisfied, then *a*) and *b*) of Proposition 3.5 clearly hold because *D* and $\sqrt{1 + D^{2}}$ are bounded. $\quad \square$

#### Finite codimensional subspaces of standard subspaces

Let *H* be a standard subspace of the complex Hilbert space ${{\mathcal {H}}}$ and $\dot{H}\subset H$ a finite-codimensional closed subspace of *H*.

With *D* and $\dot{D}$ the polarisers of *H* and $\dot{H}$, we clearly have

49 $$\begin{aligned} \dot{D} = F D|_{\dot{H}}, \end{aligned}$$

where $F : H\rightarrow \dot{H}$ is the orthogonal projection.

Let $\dot{H}^\perp \subset H$ be the real orthogonal complement of $\dot{H}$ in *H*. We have the matrix decomposition of *D* w.r.t. $H = \dot{H} + \dot{H}^\perp$

50 $$\begin{aligned} D = \left[ \begin{matrix} {\dot{D}} &{} \quad *\\ * &{}\quad * \end{matrix}\right] , \end{aligned}$$

where the starred entries have finite rank or co-rank.

##### Lemma 3.7

$1 + D^2_H \in \mathcal{L}^p(H)$ (resp. is compact) iff $1 + D^2_{\dot{H}} \in \mathcal{L}^p(\dot{H})$ (resp. is compact).

##### Proof

We have

$$\begin{aligned} (1 + D^2_H)|_{\dot{H}}= & {} 1|_{\dot{H}} + D^2_H|_{\dot{H}} = 1|_{\dot{H}} + FD_H F D_H|_{\dot{H}}\\= & {} F 1_{\dot{H}} + FD^2_H|_{\dot{H}} + \big ( FD_H (1 -F) D_H|_{\dot{H}}\big )\\= & {} 1 + D^2_{\dot{H}} + \big ( FD_H (1 -F) D_H|_{\dot{H}}\big ) \end{aligned}$$

and we may apply next lemma because $FD_H (1 -F) D_H|_{\dot{H}}$ is a finite rank operator. $\quad \square$

##### Lemma 3.8

Let ${\dot{H}}\subset H$ be a finite codimensional inclusion of Hilbert spaces, $F_k: H\rightarrow {\dot{H}}$ bounded projections and $D_k$ bounded linear operators on *H*, $k=1,2$.

Then $F_1 D_1|_{\dot{H}} - F_2 D_2|_{\dot{H}}\in \mathcal{L}^p(\dot{H})$ (resp. is compact) iff $D_1 - D_2\in \mathcal{L}^p(H)$ (resp. is compact), $p\ge 1$.

##### Proof

Suppose that $F_1 D_1|_{\dot{H}} - F_2 D_2|_{\dot{H}}$ is compact (resp. $\mathcal{L}^p$). Similarly as in (50), we have

$$\begin{aligned} D_k = F_k D_k F_k \ +\ \text {finite rank operator}, \end{aligned}$$

thus

$$\begin{aligned} D_1 - D_2 = F_1 D_1 F_1 - F_2 D_2 F_2 \ +\ \text {finite rank operator}, \end{aligned}$$

hence

$$\begin{aligned} (D_1 - D_2)|_{\dot{H}} = F_1 D_1|_{\dot{H}} - F_2 D_2 |_{\dot{H}} \ +\ \text {finite rank operator} \end{aligned}$$

is compact (resp. $\mathcal{L}^p$) by the assumption. Therefore $(D_1 - D_2)F_1$ is compact (resp. $\mathcal{L}^p$) because $F_1$ is bounded, so

$$\begin{aligned} D_1 - D_2 = (D_1 - D_2)F_1 + (D_1 - D_2)(1 - F_1) \end{aligned}$$

is compact (resp. $\mathcal{L}^p$) because $1 - F_1$ has finite rank.

The converse holds too by reversing the implications. $\quad \square$

### Local automorphisms

Let now $H_k$ be standard factorial subspaces of the Hilbert spaces ${{\mathcal {H}}}_k$, $k=1,2$ and $T: H_1 \rightarrow H_2$ a symplectic bijection, namely *T* is real linear, invertible and $\beta _2(Th, Tk) = \beta _1(h,k)$, $h,k\in H_1$, with $\beta _k$ the symplectic form on $H_k$ (the restriction of $\Im (\cdot , \cdot )_k$ to $H_k$, with $(\cdot , \cdot )_k$ the scalar product on ${{\mathcal {H}}}_k$). Then *T* promotes to a $^*$-isomorphism $\vartheta _T$ between the Weyl $C^*$-algebras $C^*(H_1)$ and $C^*(H_2)$

$$\begin{aligned} \vartheta _T\big (V_1(h)\big ) = V_2(Th). \end{aligned}$$

With ${{\mathcal {A}}}_k(H_k)$ the von Neumann algebra associated with $H_k$ on the Bose Fock space $e^{{{\mathcal {H}}}_k}$, we want to study when $\vartheta _T$ extends to a normal isomorphism between ${{\mathcal {A}}}_1(H_1)$ and ${{\mathcal {A}}}_2(H_2)$.

Let ${\widetilde{T}}: {{\mathcal {H}}}_1\rightarrow {{\mathcal {H}}}_2$ be the real linear operator, with domain $D({\widetilde{T}}) = H _1+ H'_1$ and range ${{\,\mathrm{ran}\,}}({\widetilde{T}}) = H_2 + H'_2$,

$$\begin{aligned} {\widetilde{T}}: h + J_1 k \mapsto Th + J_2 Tk,\quad h,k\in H_1, \end{aligned}$$

where $H'_k$ is the symplectic complement of $H_k$ in ${{\mathcal {H}}}_k$ and $J_k = J_{H_k}$. Then ${\widetilde{T}}$ is a densely defined, real linear, symplectic map with dense range from ${{\mathcal {H}}}_1$ to ${{\mathcal {H}}}_2$.

#### Lemma 3.9

If ${\widetilde{T}}i_1 - i_2 {\widetilde{T}}$ is bounded and densely defined, then ${\widetilde{T}}$ is bounded.

#### Proof

${\widetilde{T}}$ is closable by Lemma 3.1 so ${\widetilde{T}} i_1$ and $i_2 {\widetilde{T}}$ are closable too. By assumptions, there is a bounded, everywhere defined operator $C:{{\mathcal {H}}}_1\rightarrow {{\mathcal {H}}}_2$ such that ${\widetilde{T}} i_1 = i_2 {\widetilde{T}} + C$ on ${{\mathcal {D}}}\equiv D({\widetilde{T}} i_1 - i_2 {\widetilde{T}})$, so the closures of ${\widetilde{T}} i_1|_{{\mathcal {D}}}$ and $i_2 {\widetilde{T}}|_{{\mathcal {D}}}$ have the same domain. Now

$$\begin{aligned} {{\mathcal {D}}}= D({\widetilde{T}}) \cap i_1 D({\widetilde{T}}) = D(P_{H_1}) \cap i_1 D(P_{H_1}) \end{aligned}$$

is a core for $P_{H_1}$, as follows by Eq. (12). Indeed, $\Delta _{i_1 H_1} = \Delta _{H_1}$ and $J_{i_1 H_1} = - J_{H_1}$, so the spectral subspaces of $\Delta _{H_1}$ relative to finite closed intervals $[a,b]\subset (0,1)\cup (1,\infty )$ are in the domain of $D(P_{H_1})\cap D(P_{i_1{H_1}})$ (see [9]).

Now,

$$\begin{aligned} {\widetilde{T}} = T P_{H_1} + J_2TJ_1(1 - P_{H_1}) \end{aligned}$$

and one easily checks that ${{\mathcal {D}}}$ is a core for ${\widetilde{T}}$, similarly as above. It follows that $\bar{{\widetilde{T}}}i_1 = i_2\bar{{\widetilde{T}}} + C$, with $\bar{{\widetilde{T}}}$ the closure of ${\widetilde{T}}$. Therefore, $D(\bar{{\widetilde{T}}}i_1)= D(i_2\bar{{\widetilde{T}}})$, so $i_1 D(\bar{{\widetilde{T}}}) = D(\bar{{\widetilde{T}}})$. We conclude that

$$\begin{aligned} D(\bar{{\widetilde{T}}})\supset (H_1 + H_1')+ i_1(H_1 + H_1')\supset H_1 + i_1H_1' = H_1 + H_1^\perp = {{\mathcal {H}}}_1, \end{aligned}$$

so ${\widetilde{T}}$ is bounded by the closed graph theorem. $\quad \square$

#### Proposition 3.10

The following are equivalent:

(i) There exists a unitary $U: e^{{{\mathcal {H}}}_1}\rightarrow e^{{{\mathcal {H}}}_2}$ such that $UV_1(h) U^* = V_2(Th)$, $h \in H_1$;
(ii) $\vartheta _T$ extends to a normal isomorphism ${{\mathcal {A}}}_1(H_1) \rightarrow {{\mathcal {A}}}_2(H_2)$;
(iii) $${\widetilde{T}}^* {\widetilde{T}} - 1 \in \mathcal{L}^2({{\mathcal {H}}}_1)$$;
(iv) $${\widetilde{T}}i_1 - i_2 {\widetilde{T}}\in \mathcal{L}^2({{\mathcal {H}}}_1,{{\mathcal {H}}}_2)$$.

#### Proof

$\mathrm{(i)} \Leftrightarrow \mathrm{(ii)}$: Clearly (ii) follows from (i); we show that $\mathrm{(ii)} \Rightarrow \mathrm{(i)}$. Let $V_k(\cdot )$ be the Weyl unitary on $e^{{{\mathcal {H}}}_k}$. By assumptions, the linear extension of the map $V_1(h)\mapsto V_2(Th)$, $h\in H_1$, extends to a normal isomorphism ${\bar{\vartheta }}_T:{{\mathcal {A}}}_1(H_1)\rightarrow {{\mathcal {A}}}_2(H_2)$. Since the vacuum vector is cyclic and separating for ${{\mathcal {A}}}_k(H_k)$, we have the associated unitary standard implementation $U_T: e^{{{\mathcal {H}}}_1}\rightarrow e^{{{\mathcal {H}}}_2}$ of ${\bar{\vartheta }}_T$ w.r.t. the vacuum vectors [3, 11, 21].

$\mathrm{(i)} \Leftrightarrow \mathrm{(iii)}$: Assume (i) and let $U_T$ be the vacuum unitary standard implementation ${\bar{\vartheta }}_T$ as above. $e^{J_k}$, the second quantisation of the modular conjugation $J_k$ of $H_k$, is the modular conjugation of the von Neumann algebra ${{\mathcal {A}}}_k(H)$ w.r.t. the vacuum vector $e^0$, so we have

$$\begin{aligned} U_T V_1(h) U_T^* = V_2(Th),\quad U_T e^{J_1} = e^{J_2} U_T , ,\quad h\in H_1, \end{aligned}$$

therefore

$$\begin{aligned} U_T V_1(h) V_1(J_1 k) U_T^* = V_2(h) V_2(J_2 k),\quad h,k\in H_1, \end{aligned}$$

namely

$$\begin{aligned} U_T V_1(h + J_1 k) U_T^* = V_2(Th + J_2 Tk), \end{aligned}$$

that is

51 $$\begin{aligned} \ U_T V_1(\eta ) U_T^* = V_2({\widetilde{T}}\eta ), \end{aligned}$$

for all $\eta$ in the domain of ${\widetilde{T}}$. Then (iii) holds by Lemma 3.1 and Shale’s criterion [39]. Conversely, assuming (iii), by Lemma 3.9 and again by Lemma 3.1 and Shale’s criterion, we can find a unitary *U* such that (51) holds.

(iii) and (iv) are equivalent, by using Lemmas 3.1 and 3.9, see e.g. [30]. $\quad \square$

#### Corollary 3.11

Let $T: H_1\rightarrow H_2$ be a symplectic bijection. Then the Bogoliubov isomorphism $\vartheta _T: A(H_1) \rightarrow A(H_2)$ is implemented by a unitary $U: e^{{{\mathcal {H}}}_1} \rightarrow e^{{{\mathcal {H}}}_2}$ iff the following conditions hold:

$$\begin{aligned}&\mathrm{(a)}\ \Big (TD_1^{-1} - D_2^{-1}T\Big ) - \sqrt{1 + D_2^{2}}\,\Big (TD_1^{-1}\sqrt{1 + D_1^{2}} - D_2^{-1}\sqrt{1 + D_2^{2}}\, T\Big ) \in \mathcal{L}^2(H_1, H_2)\\&\mathrm{(b)}\ D_2\Big (TD_1^{-1}\sqrt{1 + D_1^{2}}- D_2^{-1}\sqrt{1 + D_2^{2}}\, T\Big ) \in \mathcal{L}^2(H_1, H_2). \end{aligned}$$

#### Proof

The above conditions are the straightforward generalisations of the conditions *a*) and *b*) in Proposition 3.5, so the corollary follows by Proposition 3.10. $\quad \square$

Recall that a real linear map $T: H_1 \rightarrow H_2$ is symplectic iff $T^*D_2 = D_1 T^{-1}$, so the conditions in the above corollary take a different form by inserting this relation.

## Gaussian States, Modular Hamiltonian, Quasi-equivalence

Let $(H,\beta )$ be a symplectic space. With $\alpha$ a real scalar product on *H* compatible with $\beta$, let $\kappa _\alpha : H\rightarrow {{\mathcal {H}}}_\alpha$ be the one-particle structure associated with $\alpha$ (Proposition 2.1).

Let $e^{{{\mathcal {H}}}_\alpha }$ be the Bose Fock Hilbert space over ${{\mathcal {H}}}_\alpha$ and denote by $V_\alpha (\cdot )$ the Weyl unitaries acting on $e^{{{\mathcal {H}}}_\alpha }$ and by $e^0$ the vacuum vector of $e^{{{\mathcal {H}}}_\alpha }$, thus $V(h)\mapsto V_\alpha (h)$ gives a representation of $C^*(H)$ on $e^{{{\mathcal {H}}}_\alpha }$ (see for example [26]). By (31), we have

52 $$\begin{aligned} (e^0, V_\alpha (\kappa _\alpha (h))e^0) = e^{-\frac{1}{2} ||\kappa _\alpha (h)||^2} = e^{-\frac{1}{2} \alpha (h,h)} ,\quad h\in H. \end{aligned}$$

### Proposition 4.1

There exists a unique state $\varphi _\alpha$ on $C^*(H)$ such that

53 $$\begin{aligned} \varphi _\alpha \big (V(h)\big ) = e^{-\frac{1}{2} \alpha (h,h)}. \end{aligned}$$

With $\{{{\mathcal {H}}}_{\varphi _\alpha }, \pi _{\varphi _\alpha }, \xi _{\varphi _\alpha }\}$ the GNS triple associated with $\varphi _\alpha$, the vector $\xi _{\varphi _\alpha }$ is separating for the von Neumann algebra ${{\mathcal {A}}}(H)=\pi _{\varphi _\alpha }\big (C^*(H)\big )''$ iff the completion ${\bar{H}}$ of *H* is a separating subspace, namely $\ker (D_{{\bar{H}}}^2 + 1) = \{0\}$.

### Proof

Equation (52) shows that there exists a state $\varphi _a$ such that (53) holds. Moreover (53) determines $\varphi _\alpha$ because the linear span of the Weyl unitaries is a dense subalgebra of $C^*(H)$.

As $\kappa _\alpha (H)$ is cyclic in ${{\mathcal {H}}}_\alpha$, $\overline{\kappa _\alpha (H)}$ is a standard subspace of ${{\mathcal {H}}}_\alpha$ iff $\overline{\kappa _\alpha (H)}$ is separating. On the other hand, $e^0$ is cyclic and separating for the von Neumann algebra generated by the $V_\alpha (h)$’s, $h\in H$, iff $\overline{\kappa _\alpha (H)}$ is a standard subspace of ${{\mathcal {H}}}$, see [26]. The proposition then follows by the uniqueness of the GNS representation. $\quad \square$

The state $\varphi _\alpha$ determined by (53) is well known and is called the *Gaussian*, or *quasi-free, state* associated with $\alpha$, see [14, 34]. It is usually defined by showing directly, by positivity, that the Gaussian kernel (53) defines a state.

We summarise in the following diagram the two above considered, unitarily equivalent constructions with the GNS representation of a Gaussian state: 
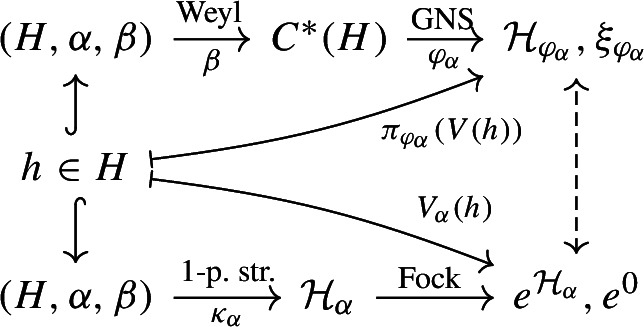
 As a consequence, if *H* is a standard subspace, the modular group $\sigma ^{\varphi _\alpha }$ of $\varphi _\alpha$ on $C^*(H)$ is given by

$$\begin{aligned} \sigma _s^{\varphi _\alpha }\big (V(h)\big ) = V\big (\Delta _H^{is}h\big ),\quad h\in H, \ s\in {{\mathbb {R}}}, \end{aligned}$$

therefore the study of the modular structure of ${{\mathcal {A}}}(H)$ can be reduced to the study of the modular structure of *H*.

The following quasi-equivalence criterion is related to the analysis in [5, 23, 42], although we do not rely on their work.

In the following, we shall always deal with *factorial standard subspaces*.

### Theorem 4.2

Let $(H, \alpha _k, \beta )$ be factorial, abstract standard subspaces, $k=1,2$. The Gaussian states $\varphi _{\alpha _1}$ and $\varphi _{\alpha _2}$ are quasi-equivalent iff both

54 $$\begin{aligned} (D^{-1}_1 - D^{-1}_2) - \sqrt{1 + D_2^{2}}\,\Big (D^{-1}_1\sqrt{1 + D_1^{2}} - D^{-1}_2\sqrt{1 + D_2^{2}}\Big ) \in \mathcal{L}^2(H) \end{aligned}$$

and

55 $$\begin{aligned} D_2 \Big (D^{-1}_1\sqrt{1 + D_1^{2}} - D^{-1}_2\sqrt{1 + D_2^{2}}\Big ) \in \mathcal{L}^2(H), \end{aligned}$$

hold, where $D_k$ is the polariser of $(H, \alpha _k, \beta )$.

### Proof

Let ${{\mathcal {H}}}_k$ be the symplectic dilation of $(H, \beta _k)$ with respect to $\alpha _k$; so $H\subset {{\mathcal {H}}}_k$ is a factorial standard subspace. We have spelled out the conditions for the symplectic map $I: {\hat{H}} \rightarrow {\hat{H}}$ to promote a unitary between the Fock spaces over ${{\mathcal {H}}}_1$ and ${{\mathcal {H}}}_2$ (*I* is the identity on $H\oplus H$ as vector spaces). Shale’s criterion gives

$$\begin{aligned} I i_1 - i_2 I \in \mathcal{L}^2({{\mathcal {H}}}_1 , {{\mathcal {H}}}_2), \end{aligned}$$

that entails the statement of the theorem by Proposition 3.5. $\quad \square$

We now consider the property

56 $$\begin{aligned} P_1 i_1|_H - P_2 i_2|_H \in \mathcal{L}^2(H), \end{aligned}$$

that is

57 $$\begin{aligned} D^{-1}_1 - D^{-1}_2 \in \mathcal{L}^2(H), \end{aligned}$$

that is

58 $$\begin{aligned} i_1\coth (L_1/2)|_H - i_2\coth (L_2/2)|_H \in \mathcal{L}^2(H). \end{aligned}$$

We write $\alpha _1 \approx \alpha _2$ if Property (56) holds.

### Corollary 4.3

Assume $\alpha _1\approx \alpha _2$. The Gaussian states $\varphi _{\alpha _1}$ and $\varphi _{\alpha _2}$ are quasi-equivalent iff

59 $$\begin{aligned} D_2^{-1}\sqrt{1 + D_2^{2}}\,\Big (\sqrt{1 + D_1^{2}} - \sqrt{1 + D_2^{2}}\Big )\in \mathcal{L}^2(H) \end{aligned}$$

and

60 $$\begin{aligned} \Big (\sqrt{1 + D_1^{2}} - \sqrt{1 + D_2^{2}}\Big )\in \mathcal{L}^2(H). \end{aligned}$$

### Proof

As $\alpha _1\approx \alpha _2$, i.e. $D^{-1}_1 - D^{-1}_2 \in \mathcal{L}^2(H)$, clearly (54) is equivalent to

61 $$\begin{aligned} \sqrt{1 + D_2^{2}}\,\Big (D^{-1}_1\sqrt{1 + D_1^{2}} - D^{-1}_2\sqrt{1 + D_2^{2}}\Big )\in \mathcal{L}^2(H), \end{aligned}$$

which is equivalent to (59).

On the other hand, (55) is equivalent to (60), again because $D^{-1}_1 - D^{-1}_2 \in \mathcal{L}^2(H)$. So the corollary follows by Thm. 4.2. $\quad \square$

### Corollary 4.4

Assume $\alpha _1\approx \alpha _2$. The Gaussian states $\varphi _{\alpha _1}$ and $\varphi _{\alpha _2}$ are quasi-equivalent iff

62 $$\begin{aligned} \Big (D_1^{-1}\sqrt{1 + D_1^{2}} - D_2^{-1}\sqrt{1 + D_2^{2}}\Big )\in \mathcal{L}^2(H) \end{aligned}$$

and

63 $$\begin{aligned} \Big (\sqrt{1 + D_1^{2}} - \sqrt{1 + D_2^{2}}\Big )\in \mathcal{L}^2(H). \end{aligned}$$

### Proof

Note first that, by (20), (63) is the same as

64 $$\begin{aligned} \frac{1}{\cosh (L_1/2)}\Big |_H - \frac{1}{\cosh (L_2/2)}\Big |_H\in \mathcal{L}^2(H). \end{aligned}$$

Let us now assume that $\alpha _1\approx \alpha _2$ and that (64) holds. By Cor. 4.3, we have to prove that (59) is equivalent to (62).

By (37), (59) is equivalent to

$$\begin{aligned} P'_2 i_2\Big (\frac{1}{\cosh (L_1/2)}\Big |_H - \frac{1}{\cosh (L_2/2)}\Big |_H\Big )\in \mathcal{L}^2(H,{{\mathcal {H}}}_2), \end{aligned}$$

with $P'_2$ the cutting projection ${{\mathcal {H}}}_2\rightarrow H$. As $P'_2 = 1 -P_2$, Eq. (59) is thus equivalent to

65 $$\begin{aligned} P_2 i_2\Big (\frac{1}{\cosh (L_1/2)}\Big |_H - \frac{1}{\cosh (L_2/2)}\Big |_H\Big )\in \mathcal{L}^2(H) , \end{aligned}$$

namely

66 $$\begin{aligned} \Big (D_2^{-1}\sqrt{1 + D_1^{2}} - D_2^{-1}\sqrt{1 + D_2^{2}}\Big )\in \mathcal{L}^2(H). \end{aligned}$$

Since $\sqrt{1 + D_1^{2}}$ is bounded, and $\alpha _1\approx \alpha _2$, the above equation is equivalent to (62). $\quad \square$

### Corollary 4.5

The Gaussian states $\varphi _{\alpha _1}$ and $\varphi _{\alpha _2}$ are quasi-equivalent if

67 $$\begin{aligned} i_1\frac{1}{\sinh (L_1/2)}\Big |_H - i_2\frac{1}{\sinh (L_2/2)}\Big |_H\in \mathcal{L}^2(H). \end{aligned}$$

### Proof

Assume first that $\alpha _1\approx \alpha _2$. Then (67), i.e. (62), is equivalent to (66), and (66) implies (63) since $D_2$ is bounded. So Cor. 4.4 applies and $\varphi _{\alpha _1}$ and $\varphi _{\alpha _2}$ are quasi-equivalent.

To end our proof, we now show that (67) implies $\alpha _1\approx \alpha _2$. Let *F* be defined by $f(x) = F\big (g(x)\big )$, with $f(x) = \coth (x)$, $g(x) = 1/\sinh (x)$. Then $f'(x) = F'(y)g'(x)$, with $y = g(x)$, so $F'(y) = f'(x)/g'(x) = (1/\sinh ^2(x))\big /(\cosh (x)/\sinh ^2(x)) = 1/\cosh (x)$, therefore *F* is uniformly Lipschitz. Since 0 is not in the point spectrum of $L_k$, it follows by Cor. 6.5 that (67) implies (58), namely $\alpha _1\approx \alpha _2$. $\quad \square$

Now, if $A_1, A_2$ are bounded, real linear operators on *H* with trivial kernel, we have

$$\begin{aligned} A_1 - A_2 = A_1(A^{-1}_2 - A^{-1}_1) A_2 \end{aligned}$$

on the domain of the right hand side operator, thus

68 $$\begin{aligned} A^{-1}_1 - A^{-1}_2 \in \mathcal{L}^p(H) \Rightarrow A_1 - A_2 \in \mathcal{L}^p(H), \quad p \ge 1 . \end{aligned}$$

We then have:

### Corollary 4.6

If

69 $$\begin{aligned} i_1\coth (L_1/4)|_H - i_2\coth (L_2/4)|_H \in \mathcal{L}^2(H), \end{aligned}$$

then the Gaussian states $\varphi _{\alpha _1}$ and $\varphi _{\alpha _2}$ on $C^*(H)$ are quasi-equivalent.

### Proof

By assumption (69) holds, so also

70 $$\begin{aligned} i_1\tanh (L_1/4) i_1 |_H - i_2\tanh (L_4/2) i_2 |_H \in \mathcal{L}^2(H), \end{aligned}$$

holds by (68); therefore

$$\begin{aligned} i_1\big (\coth (L_1/4)|_H - \tanh (L_1/4)|_H\big ) - i_2\big (\coth (L_2/4)|_H - \tanh (L_2/4)|_H\big ) \in \mathcal{L}^2(H). \end{aligned}$$

Since $\coth (x/2) - \tanh (x/2) = 2/\sinh (x)$, we have

71 $$\begin{aligned} i_1\frac{1}{\sinh (L_1/2)}\Big |_H - i_2\frac{1}{\sinh (L_2/2)}\Big |_H\in \mathcal{L}^2(H). \end{aligned}$$

So our corollary follows by Cor. 4.5. $\quad \square$

The above corollary suggests that $\varphi _{\alpha _1}$ and $\varphi _{\alpha _2}$ are quasi-equivalent if $P_1 i_1|_H - P_2 i_2|_H$ is compact with proper values decaying sufficiently fast.

### Weakly inner Bogoliubov automorphisms

In this section, we study the condition for a real linear, symplectic bijection of a standard space to give rise to a weakly inner automorphism in the representation associated with a given Gaussian state.

Let $H\subset {{\mathcal {H}}}$ be a factorial standard subspace of the complex Hilbert space ${{\mathcal {H}}}$, $T: H\rightarrow H$ a symplectic bijection and $\vartheta _T$ the associated Bogoliubov automorphism of the Weyl algebra *A*(*H*). Denote by ${{\mathcal {A}}}(H)$ the weak closure of *A*(*H*) on $e^{{\mathcal {H}}}$ as in previous sections.

We consider the real linear map on ${{\mathcal {H}}}$ given by

$$\begin{aligned} {\hat{T}}(h + h') = Th + h', \quad h\in H,\, h'\in H', \end{aligned}$$

thus $D({\hat{T}}) = \mathrm{ran}({\hat{T}}) = H + H'$. One immediately sees that ${\hat{T}}$ is a symplectic map on ${{\mathcal {H}}}$.

Note that $D([{\hat{T}}, i]) = D({\hat{T}})\cap iD({\hat{T}}) = D(P_H)\cap D(P_{iH})$ is dense in ${{\mathcal {H}}}$, indeed a core for $P_H$, as in the proof of Lemma 3.9.

#### Lemma 4.7

Let *T* be a symplectic bijection on *H*. The following are equivalent:

(i) $\vartheta _T$ extends to an inner automorphism of ${{\mathcal {A}}}(H)$;
(ii) $${\hat{T}}^*{\hat{T}} - 1\in \mathcal{L}^2({{\mathcal {H}}})$$;
(iii) $$[{\hat{T}}, i]\in \mathcal{L}^2({{\mathcal {H}}})$$.

#### Proof

Since ${{\mathcal {A}}}(H')$ is the commutant of ${{\mathcal {A}}}(H)$, $\vartheta _T$ extends to an inner automorphism of ${{\mathcal {A}}}(H)$ if and only if the Bogoliubov automorphism associated with ${\hat{T}}$ is unitarily implementable on $e^{{\mathcal {H}}}$. Therefore the equivalence $\mathrm{(i)} \Leftrightarrow \mathrm{(ii)}$ follows by Shale’s criterion and Lemma 3.1.

$\mathrm{(ii)} \Leftrightarrow \mathrm{(iii)}$ follows again by Shale’s criterion, Lemma 3.1 and the obvious adaptation of Lemma 3.9. $\quad \square$

Set now $T = 1 + X$ and ${\hat{X}} = X \oplus 0$ on $H + H'$. In the symplectic matrix decomposition, we have

$$\begin{aligned}&{\hat{X}} i = \begin{bmatrix} X D^{-1}&{}\quad X D^{-1}\sqrt{1 + D^{2}} J\\ 0 &{}\quad 0 \end{bmatrix}, \\&i{\hat{X}} = \begin{bmatrix} D^{-1}X&{}\quad 0 \\ - J D^{-1}\sqrt{1 + D^{2}}\, X&{}\quad 0 \end{bmatrix}, \\&[{\hat{T}},i] = [{\hat{X}},i]= \begin{bmatrix} [X,D^{-1}]&{}\quad X D^{-1}\sqrt{1 + D^{2}} J\\ J D^{-1}\sqrt{1 + D^{2}}X &{}\quad 0 \end{bmatrix}, \end{aligned}$$

With $C = [{\hat{X}},i]$, we apply Lemma 3.3. Then

72 $$\begin{aligned}&E_H C |_H = C_{11} + \sqrt{1 + D^{2}}\, J C_{21} = [X,D^{-1}] + (D^{-1} + D)X, \end{aligned}$$

73 $$\begin{aligned}&E_{H} C i|_{H'} =D C_{12} = D X D^{-1}\sqrt{1 + D^{2}}J, \end{aligned}$$

74 $$\begin{aligned}&E_{H'} i C |_H = J D J C_{21} = J \sqrt{1 + D^{2}}X , \end{aligned}$$

75 $$\begin{aligned}&E_{H'} C |_{H'} = J \sqrt{1 + D^{2}}\, C_{12} + C_{22} = J \sqrt{1 + D^{2}}\, X D^{-1}\sqrt{1 + D^{2}} J . \end{aligned}$$

Note that

$$\begin{aligned} D^{-1} + D= & {} - i\big (\coth (L/2) - \tanh (L/2)\big ) \big |_H \\= & {} - i/\cosh (L/2)\sinh (L/2) \big |_H = - 2i/\sinh (L)\big |_H , \\ D^{-1}\sqrt{1 + D^{2}}= & {} - i \frac{1}{\sinh (L/2)}\Big |_H. \end{aligned}$$

#### Proposition 4.8

$[{\hat{T}},i] \in \mathcal{L}^2({{\mathcal {H}}})$ iff all the operators

$$\begin{aligned}&[X,D^{-1}] + (D^{-1} + D)X = XD^{-1} + DX ,\\&D X D^{-1}\sqrt{1 + D^{2}},\\&\sqrt{1 + D^{2}}X,\\&\sqrt{1 + D^{2}}\, X D^{-1}\sqrt{1 + D^{2}} , \end{aligned}$$

are in $$\mathcal{L}^2(H)$$.

In particular, this is the case if $X D^{-1} \in \mathcal{L}^2(H)$.

#### Proof

$[{\hat{T}},i] \in \mathcal{L}^2({{\mathcal {H}}})$ iff all the operators in (72), (73), (74), (75) are Hilbert–Schmidt, so the first part of the statement holds. Now, $X D^{-1} \in \mathcal{L}^2(H)$ implies that all the operators in the statement are Hilbert–Schmidt too as they are obtained by left/right multiplication of $X D^{-1}$ by bounded operators, $X D^{-1} \in \mathcal{L}^2(H)$ is a sufficient condition for $[{\hat{T}},i] \in \mathcal{L}^2({{\mathcal {H}}})$. $\quad \square$

#### Theorem 4.9

Let $(H,\alpha ,\beta )$ be an abstract factorial standard subspace and $T : H\rightarrow H$ a bijective symplectic map. Then $\vartheta _T$ extends to an inner automorphism of the von Neumann algebra ${{\mathcal {A}}}(H)$, in the GNS representation of $\varphi _\alpha$ iff the conditions in Proposition 4.8 hold.

#### Proof

The theorem follows now by Lemma (4.7). $\quad \square$

## QFT and the Modular Hamiltonian

We now work out the studied abstract structure, within the context of Quantum Field Theory. We then provide a couple of applications of our results.

### One-particle space of the free scalar QFT

This section concerns the one-particle space of the free scalar QFT, especially in the low dimensional case. Although we are primarily interested in the low dimensional case in this paper, we start by describing the higher dimensional case in order to clarify the general picture. In the following, *d* is the space dimension, so ${{\mathbb {R}}}^d$ is the time-zero space of the Minkowski spacetime ${{\mathbb {R}}}^{d+1}$, cf. [30].

#### Case $d\ge 2, m\ge 0$

Let ${{\mathcal {S}}}$ denote the real linear space of smooth, compactly supported real functions on ${\mathbb {R}}^d$, $d\ge 2$.

Let $H_m^{\pm 1/2}$ be the real Hilbert space of real tempered distributions $f \in S'({{\mathbb {R}}}^d)$ such that the Fourier transform ${\hat{f}}$ is a Borel function and

76 $$\begin{aligned} ||f ||_{\pm 1/2}^2 = \int _{{{\mathbb {R}}}^d} {(|\mathbf{p}|^2 + m^2)}^{\pm 1/2} | {\hat{f}}(\mathbf{p})|^2 d\mathbf{p}< +\infty . \end{aligned}$$

${{\mathcal {S}}}$ is dense in $H_m^{\pm 1/2}$ and $\mu _m : H_m^{1/2} \rightarrow H_m^{-1/2}$, with

77 $$\begin{aligned} \widehat{\mu _m f}({\mathbf{p}}) = \sqrt{{|\mathbf{p}|}^2 + m^2}\,{\hat{f}}({\mathbf{p}}), \end{aligned}$$

is a unitary operator. Then

78 $$\begin{aligned} \imath _m = \left[ \begin{matrix} 0 &{}\quad \mu _m^{-1} \\ -\mu _m &{}\quad 0 \end{matrix}\right] \end{aligned}$$

is a unitary operator $\imath _m$ on $H_m = H_m^{1/2}\oplus H_m^{-1/2}$ with $\imath _m^2 = -1$, namely a complex structure on $H_m$ that so becomes a complex Hilbert space ${{\mathcal {H}}}_m$ with the imaginary part of the scalar product given by

79 $$\begin{aligned} \Im ( \langle f,g\rangle , \langle h,k \rangle )_m = \frac{1}{2}\big ( (h, g)-(f,k)\big ) , \end{aligned}$$

which is independent of $m\ge 0$ (where $(\cdot , \cdot )$ is the $L^2$ scalar product).

With *B* the unit ball of ${\mathbb {R}}^{d}$, we shall denote by $H^{\pm 1/2}_m(B)$ the subspace of $H^{\pm 1/2}_m$ associated with *B* consisting of the distributions $f\in S'({{\mathbb {R}}}^d)$ as above that are supported in *B*. We have

$$\begin{aligned} H^{\pm 1/2}_m(B) = \text {closure of }C_0^\infty (B)\text { in }H^{\pm 1/2}_m, \end{aligned}$$

and the standard subspace of ${{\mathcal {H}}}_m$ associated with *B* is

$$\begin{aligned} H_m(B) \equiv H_m^{1/2}(B)\oplus H_m^{-1/2}(B). \end{aligned}$$

Here $C^{\infty }_0(B)$ denotes the space of real $C^\infty$ function on ${\mathbb {R}}^d$ with compact support in *B*.

The $H_m(B)$’s, $m\ge 0$, are the same linear space with the same Hilbert space topologies (see e.g. [30]). We shall often identify these spaces as topological vector spaces.

In the following, we consider the abstract standard spaces $(H,\alpha _m, \beta )$ where $H= H_m(B)$, $\beta$ is the symplectic form on *H* given by (79) and $\alpha _m$ is the real scalar product on *H* as a real subspace of ${{\mathcal {H}}}_m$.

Denote by $P_m$ the cutting projection on ${{\mathcal {H}}}_m$ relative to $H_m(B)$. Then $P_m \imath _m |_{H_m(B)}$ is a real linear, densely defined operator on *H*.

##### Proposition 5.1

$$P_m \imath _m|_{H^{1/2}_m(B)} - P_0 \imath _0 |_{H^{1/2}_0(B)}$$ is $$\mathcal{L}^p(H^{1/2}_m(B),H^{-1/2}_m(B))$$ if $$p>d/2$$.

##### Proof

The cutting projection $P_m$ is given by the matrix $$\left[ \begin{matrix} P_+ &{}\quad 0 \\ 0 &{}\quad P_-\end{matrix}\right] $$, with $P_\pm : D(P_\pm ) \subset H_m^{\pm 1/2} \rightarrow H_m^{\pm 1/2}$ the operator of multiplication by the characteristic function $\chi _B$ of *B* in $H_m^{\pm 1/2}$ [9, 30]. Thus we have

$$\begin{aligned} P_m \imath _m = \left[ \begin{matrix} 0 &{}\quad P_+\mu _m^{-1} \\ - P_{\! -} \mu _m &{}\quad 0\end{matrix}\right] \end{aligned}$$

and we have to show that $P_- \mu _m - P_- \mu _0: H_m^{1/2}(B) \rightarrow H_m^{-1/2}(B)$ is in $\mathcal{L}^p$ iff $p>d/2$, namely that

$$\begin{aligned} f\in H_m^{1/2}(B)\mapsto (\mu _m - \mu _0)f |_B \in H_m^{-1/2}(B) \end{aligned}$$

is $\mathcal{L}^p$ iff $p>d/2$. Note that, in Fourier transform,

80 $$\begin{aligned} \big ((\mu _m - \mu _0)f \big ){}^{\widehat{}}(\mathbf{p}) = \big (\sqrt{|\mathbf{p}|^2 + m^2} - \sqrt{|\mathbf{p}|^2}\big ){\hat{f}}(\mathbf{p}) = \frac{m^2}{\sqrt{|\mathbf{p}|^2 + m^2} + \sqrt{|\mathbf{p}|^2}}{\hat{f}}(\mathbf{p}).\qquad \end{aligned}$$

We have the following commutative diagram 
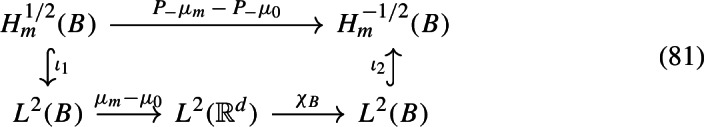
 where $\chi _B$ is the multiplication operator by the characteristic function of *B* in $L^2({{\mathbb {R}}}^d)$, i.e. the orthogonal projection $L^2({{\mathbb {R}}}^d)\rightarrow L^2(B)$, and $\iota _1$, $\iota _2$ are natural embeddings.

We need a couple of lemmas in order to conclude our proof.

##### Lemma 5.2

The operator $(\mu _m - \mu _0): L^2(B) \rightarrow L^2({{\mathbb {R}}}^d)$ is in $\mathcal{L}^p$ iff $p >d$.

##### Proof

By (80) we have

82 $$\begin{aligned} \big ((\mu _m - \mu _0)f \big ){}^{\widehat{}}(\mathbf{p}) =a(|\mathbf{p}|)\big (|\mathbf{p}|^2 + m^2\big )^{-1/2} {\hat{f}}(\mathbf{p}) \end{aligned}$$

with $a(s)= m^2 \sqrt{s^2 + m^2}/(\sqrt{s^2 + m^2} + s)$, so and 1/*a* are bounded continuous functions on ${{\mathbb {R}}}^d$. Therefore

83 $$\begin{aligned} \mu _m - \mu _0 = A \big (\nabla ^2 - m^2\big )^{-1/2}, \end{aligned}$$

with *A* the multiplication operator by *a*, a bounded linear operator with bounded inverse. So

$$\begin{aligned} \big (\nabla ^2 - m^2\big )^{-1/2}|_{L^2(B)} \in \mathcal{L}^p \Leftrightarrow (\mu _m - \mu _0)|_{L^2(B)} \in \mathcal{L}^p \end{aligned}$$

as operator $L^2(B)\rightarrow L^2({{\mathbb {R}}}^d)$. Let us show that $\mu _m^{-1}|_{L^2(B)} = \big (\nabla ^2 - m^2\big )^{-1/2}|_{L^2(B)}\in \mathcal{L}^p(L^2(B),L^2({{\mathbb {R}}}^d))$, namely that $T = \mu _m^{-1} E \in \mathcal{L}^p(L^2({{\mathbb {R}}}^d))$, with *E* the orthogonal projection $L^2({{\mathbb {R}}}^d)\rightarrow L^2(B)$. As $\mu _m^{-1}: L^2({{\mathbb {R}}}^d)\rightarrow L^2({{\mathbb {R}}}^d)$ is selfadjoint, we have $T^* = E\mu _m^{-1}$, so we have to show that $T^*T = E\mu _m^{-2}E\in \mathcal{L}^{\frac{p}{2}}$, namely that

$$\begin{aligned} E(\nabla ^2 - m^2)^{-1} |_{H_m^{1/2}(B)} \in \mathcal{L}^{\frac{p}{2}}(L^2(B)). \end{aligned}$$

Now, $E(\nabla ^2 - m^2)^{-1}$ is equal to $(\nabla _m^2 - m^2)^{-1}$, with $\nabla ^2_m$ the Laplacian on *B* with external boundary condition (6.3). We conclude that

$$\begin{aligned} E\big (\nabla ^2 - m^2\big )^{-1} |_{L^2(B)} \in \mathcal{L}^{\frac{p}{2}}(L^2(B)) \Leftrightarrow (\nabla _m^2 - m^2)^{-1} \in \mathcal{L}^{\frac{p}{2}}(L^2(B)) \Leftrightarrow p > d \end{aligned}$$

by Corollary 6.7. $$\quad \square $$

##### Lemma 5.3

Both embeddings $\iota _1: H_m^{1/2}(B)\hookrightarrow L^2(B)$ and $\iota _2: L^2(B)\hookrightarrow H_m^{-1/2}(B)$ are in $\mathcal{L}^p$ if $p >2d$. (Also if $d= 1$, $m>0$ in this lemma.)

##### Proof

By Gramsch’s result [18], the embedding $H_m^{k}(B) \hookrightarrow H_m^{l}(B)$ is in $\mathcal{L}^p$ iff $k-l > \frac{d}{p}$. In particular, $\iota _1$ and $\iota _2$ are in $\mathcal{L}^p$ iff $p > 2d$. $\quad \square$

Recall the generalised Hölder inequality for operators in the Schatten ideals: if $p\ge 1$, $p_k\ge 1$,

84 $$\begin{aligned} T_1\in \mathcal{L}^{p_1},\ T_2\in \mathcal{L}^{p_2}\ldots T_n\in \mathcal{L}^{p_n}\Rightarrow T_1 T_2\cdots T_n \in \mathcal{L}^{p} \quad \mathrm{if}\quad \frac{1}{p} = \frac{1}{p_1} + \frac{1}{p_2} + \cdots \frac{1}{p_n},\nonumber \\ \end{aligned}$$

see [40, Thm. 2.8].

##### End of proof of Proposition 5.1

We first show that $P_- \mu _m - P_-\mu _0: H_m^{1/2}(B) \rightarrow H_m^{-1/2}(B)$ is $\mathcal{L}^p$ iff $p>d/2$. This operator is the product of three operators $\iota _2[(\chi _B(\mu _m -\mu _0)]\iota _1$, see the commutative diagram (81). By Lemmas 5.2, 5.3, and by formula (84), we then get that $P_- \mu _m - P_-\mu _0: H_m^{1/2}(B) \rightarrow H_m^{-1/2}(B)$ is $\mathcal{L}^p$ if

$$\begin{aligned} \frac{1}{p} = \frac{1}{p_1} + \frac{1}{p_2} + \frac{1}{p_3} , \quad p_1> d,\ p_2> 2d,\ p_3 > 2d, \end{aligned}$$

thus if $$p > d/2$$. $$\quad \square $$

#### Case $d=1$

- *Case* $m >0$. In this case the one-particle Hilbert space is defined exactly as in the higher dimensional case. In particular $H_m^{\pm 1/2}$ is defined by (76) and $\imath _m$ (78) is a complex structure on $H_m= H^{1/2}_m\oplus H_m^{-1/2}$; so we have a complex Hilbert space ${{\mathcal {H}}}_m$, $m>0$. The subspace $H^{\pm 1/2}_m(B)$ of $H^{\pm 1/2}_m$ is again defined as in the higher dimensional case, with $B = (-1,1)$.

We now set

$$\begin{aligned} \dot{H}^{- 1/2}_m(B) = \text {closure of }\dot{C}_0^\infty (B)\text { in }H^{-1/2}_m, \end{aligned}$$

with

85 $$\begin{aligned} {\dot{{{\mathcal {S}}}}} = \Big \{f\in {{\mathcal {S}}}: {\hat{f}}(0) = \int _{{{\mathbb {R}}}} f(x)dx =0\Big \} , \end{aligned}$$

$$\dot{C}_0^\infty (B) = C_0^\infty (B)\cap {\dot{{{\mathcal {S}}}}}$$, and

86 $$\begin{aligned} \dot{H}_m(B) \equiv H_m^{1/2}(B)\oplus \dot{H}_m^{-1/2}(B) . \end{aligned}$$

##### Proposition 5.4

$\dot{H}_m(B)$ is a standard subspace of ${{\mathcal {H}}}_m$ of

87 $$\begin{aligned} {\dot{{{\mathcal {H}}}}}_m \equiv \overline{\dot{H}_m(B) + \imath _m \dot{H}_m(B)} . \end{aligned}$$

##### Proof

As $\dot{H}_m(B)\subset H_m(B)$, clearly $\dot{H}_m(B)$ is separating, so the statement is obvious. $\quad \square$

- *Case* $m= 0$. $H_0^{1/2}$ is defined as in the higher dimensional case (76):
  $$ \begin{aligned} H_0^{1/2} =\Big \{ f\in S'({{\mathbb {R}}}):{\hat{f}} \ \text {Borel function}\  \&  \int _{{{\mathbb {R}}}} |\mathbf{p}| | {\hat{f}}(\mathbf{p})|^2 d\mathbf{p}< +\infty \Big \} . \end{aligned}$$

We now set

$$ \begin{aligned} \dot{H}_0^{-1/2} = \Big \{ f\in S'({{\mathbb {R}}}): {\hat{f}} \ \text {Borel function}\  \&  \int _{{{\mathbb {R}}}} |\mathbf{p}^{-1}| | {\hat{f}}(\mathbf{p})|^2 d\mathbf{p}< +\infty \Big \} . \end{aligned}$$

Note that

$$\begin{aligned} {{\mathcal {S}}}\subset H^{\pm 1/2}_m, \ m > 0 ;\quad {{\mathcal {S}}}\subset H^{1/2}_0\, ;\quad {\dot{{{\mathcal {S}}}}}\subset \dot{H}^{-1/2}_0, \end{aligned}$$

Then $\imath _0$ (defined by (78) with $m=0$) is a complex structure on $\dot{H}_0= H^{1/2}_0\oplus \dot{H}_0^{-1/2}$ and we get a complex Hilbert space ${\dot{{{\mathcal {H}}}}}_0$ with underlying real Hilbert space $\dot{H}_0$.

The subspace $H^{ 1/2}_0(B)$ of $H^{ 1/2}_0$ is defined as in the higher dimensional case. We also set

$$\begin{aligned} \dot{H}^{- 1/2}_0(B) = \text {closure of }\dot{C}_0^\infty (B)\text { in }\dot{H}^{-1/2}_0, \end{aligned}$$

and

88 $$\begin{aligned} \dot{H}_0(B) \equiv H_0^{1/2}(B)\oplus \dot{H}_0^{-1/2}(B) . \end{aligned}$$

$\dot{H}_0(B)$ is a standard subspace of ${\dot{{{\mathcal {H}}}}}_0$. Note that, in the massless case, our *notation is unconventional:* ${\dot{{{\mathcal {H}}}}}_0$ is the usual one-particle space and ${{\mathcal {H}}}_0$ has not been defined yet. See also [6, 12] for related structures.

### The modular Hamiltonian, $d=1$

We now describe the modular Hamiltonian associated with the unit double cone in the free, scalar QFT on the $1+1$ dimensional Minkowski spacetime. Recall that the modular Hamiltonian on the Fock space is the second quantisation of the modular Hamiltonian on the one-particle space, that will therefore be the subject of our analysis. In this subsection $B = (-1,1)$.

#### Lemma 5.5

The $\dot{H}_m(B)$’s, $m\ge 0$, are the same linear space with the same Hilbert space topologies. Moreover, $\dot{H}_m(B)$ is a factorial standard subspace of ${\dot{{{\mathcal {H}}}}}_m$.

#### Proof

The proof that the natural, real linear identifications of the $\dot{H}_m(B)$’s preserve the Hilbert space topology is a simple adaptation of the one given in the higher dimensional case, see [30].

We have seen in Proposition 5.4 that $\dot{H}_m(B)$ is a standard subspace of ${\dot{{{\mathcal {H}}}}}_m$. The factoriality of $\dot{H}_0(B)$ follows, for example, by [22]. Now, the identification of $\dot{H}_m(B)$ with $\dot{H}_0(B)$ preserves the symplectic form. Since the factoriality is equivalent to the non-degeneracy of the symplectic form, also $\dot{H}_m(B)$ is factorial. $\quad \square$

#### Lemma 5.6

$\dot{H}_m(B)'$, the symplectic complement of $\dot{H}_m(B)$ in ${\dot{{{\mathcal {H}}}}}_m$, is equal to $H_m(B)'\cap {\dot{{{\mathcal {H}}}}}_m$.

#### Proof

The inclusion $H_m(B)'\cap {\dot{{{\mathcal {H}}}}}_m\subset \dot{H}_m(B)'$ is immediate. We prove the opposite inclusion. Let $f\oplus g\in {\dot{{{\mathcal {H}}}}}_m = H^{1/2}_m\oplus \dot{H}_m^{-1/2}$ belong to $\dot{H}_m(B)'$. By (79),

89 $$\begin{aligned} (h, g)-(f,k) = 0 \end{aligned}$$

for all $$h\oplus k \in \dot{H}_m(B)= H^{1/2}_m(B)\oplus \dot{H}_m^{-1/2}(B)$$.

Setting $k=0$, we see that $(h, g)=0$ for all $h\in C^\infty _0(B)$, so *g* is supported in the complement $B^c$ of *B*, so $g\in H^{-1/2}_m(B^c)$ (for example by Haag duality).

Set now $h=0$. Then $(f,k) = 0$ for all $k\in \dot{H}^{-1/2}_m(B)$. Let *F* be the bounded linear functional on $H^{-1/2}_m(B)$

$$\begin{aligned} F(k) \equiv (f,k) = \int f k,\quad k\in H^{-1/2}_m(B); \end{aligned}$$

as $\dot{H}^{-1/2}_m(B)$ has codimension one in $H^{-1/2}_m(B)$, there exists $f_0\in H^{1/2}_m(B)$ such that, in particular,

$$\begin{aligned} F(k) = \int f_0 k,\quad k\in L^2(B), \end{aligned}$$

therefore $f_0 =0$. So $(f,k) = 0$ for all $k\in C^\infty _0(B)$ and this implies $f \in H^{1/2}(B^c)$ by Haag duality. $\quad \square$

Denote by $\dot{P}_m$ the cutting projection on ${\dot{{{\mathcal {H}}}}}_m$ relative to $\dot{H}_m(B)$.

#### Lemma 5.7

We have

90 $$\begin{aligned} \dot{P}_m = \left[ \begin{matrix} P_+ &{}\quad 0 \\ 0 &{}\quad \dot{P}_-\end{matrix}\right] \end{aligned}$$

with $$P_+$$ (resp. $$\dot{P}_-$$) the operator of multiplication by $$\chi _B$$ on $$ H_m^{ 1/2}$$ (resp. on $$\dot{H}_m^{- 1/2}$$).

#### Proof

Let $f\oplus g\in {\dot{{{\mathcal {H}}}}}_m = H^{1/2}_m\oplus \dot{H}_m^{-1/2}$ be in the domain of $\dot{P}_m$ and set $\dot{P}_m (f\oplus g) = f_0\oplus g_0 \in \dot{H}_m(B)$. Thus $(f - f_0)\oplus (g - g_0)$ belongs to $\dot{H}_m(B)'$, the symplectic complement of $\dot{H}_m(B)$ in ${\dot{{{\mathcal {H}}}}}_m$; so, by Lemma 5.6,

$$\begin{aligned} (f - f_0)\oplus (g - g_0) \in H^{1/2}_m(B^c)\oplus \dot{H}^{-1/2}_m(B^c) \end{aligned}$$

and this shows that $\dot{P}_m$ is a diagonal matrix of the form (90).

We then have

$$\begin{aligned} P_- g = g_0 = \chi _B g_0 = \chi _B\big ( (g - g_0) + g_0\big ) = \chi _B g . \end{aligned}$$

The equation $P_+ f = \chi _B f$, with *f* in the domain of $P_+$, follows by similar arguments. $\quad \square$

#### Proposition 5.8

$$(\dot{P}_m \imath _m - \dot{P}_0 \imath _0) |_{\dot{H}^{-1/2}_m(B)}$$ belongs to $$\mathcal{L}^1(\dot{H}^{-1/2}_m(B), H^{1/2}_m(B))$$.

#### Proof

By Lemma 5.7, we have

$$\begin{aligned} \dot{P}_m \imath _m = \left[ \begin{matrix} 0 &{}\quad P_+\mu _m^{-1} \\ -\dot{P}_{\! -} \mu _m &{}\quad 0\end{matrix}\right] . \end{aligned}$$

We have to show that $\dot{P}_- \mu _m -\dot{P}_- \mu _0: \dot{H}_m^{1/2}(B) \rightarrow \dot{H}_m^{-1/2}(B)$ is in $\mathcal{L}^1$, namely, namely that

$$\begin{aligned} f\in \dot{H}_m^{1/2}(B)\mapsto (\mu _m - \mu _0)f |_B \in \dot{H}_m^{-1/2}(B) \end{aligned}$$

is $\mathcal{L}^1$. Similarly as above, we have the following commutative diagram 
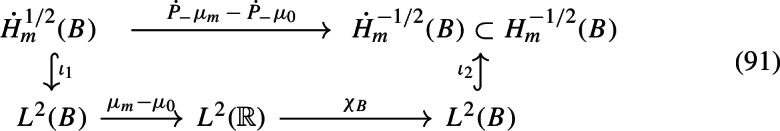


Here $\iota _1$ is the restriction to $\dot{H}_m^{1/2}(B)$ of the embedding of $H_m^{1/2}(B)$ into $L^2({{\mathbb {R}}})$. Then $\dot{P}_- \mu _m - \dot{P}_-\mu _0: \dot{H}_m^{1/2}(B) \rightarrow \dot{H}_m^{-1/2}(B)$ is $\mathcal{L}^1$ by the same argument as in the proof of Proposition 5.1. $\quad \square$

#### $m = 0$

In the massless case, the modular group associated with the unit, time-zero interval *B* acts geometrically on the spacetime double cone spanned by *B* [22]. We have:

##### Theorem 5.9

In the free scalar, massless, quantum field theory in $1+1$ spacetime dimension, the modular Hamiltonian $\log {\dot{\Delta }}_{B,0}$ associated with the unit interval *B*, that is with the standard subspace $\dot{H}_0(B)\subset {\dot{{{\mathcal {H}}}}}_0$, is given by

92 $$\begin{aligned} \log {\dot{\Delta }}_{B,0} = 2\pi \imath _0 \left[ \begin{matrix} 0 &{}\quad \frac{1}{2}(1 - x^2) \\ \frac{1}{2}(1 - x^2)\partial _x^2 - x\partial _x &{}\quad 0 \end{matrix}\right] ; \end{aligned}$$

Setting $\log {\dot{\Delta }}_{B,0} = -2\pi \dot{A}_0$ and $\dot{A}_0 \equiv -\imath _0 \dot{K}_0$, we have that $\dot{K}_0$ is essentially skew-selfadjoint on ${{\mathcal {S}}}\times {\dot{{{\mathcal {S}}}}}$. $\dot{K}_0^B = \dot{K}_0 |_{\dot{H}_0(B)}$ is skew-selfadjoint on $\dot{H}_0(B)$ and $C^\infty _0(B)\times \dot{C}^\infty _0(B)$ is a core for $\dot{K}_0^B$.

##### Proof

The formula is obtained as in [30], with obvious modifications. $\quad \square$

#### $$ m >0$$

The following analysis, done in [30] in the case $d\ge 2$, extends verbatim to the case $d=1$. Let $K_m^B :D(K_m^B)\subset H_m(B)\rightarrow H_m(B)$ be the real linear operator on $H_m(B)$ given by

93 $$\begin{aligned} K_m^B = \left[ \begin{matrix} 0 &{}\quad \frac{1}{2}(1 - r^2) \\ \frac{1}{2}(1 - r^2)(\nabla ^2 -m^2) - r\partial _r - \frac{1}{2} m^2 G^B_m&{}\quad 0 \end{matrix}\right] \end{aligned}$$

($m>0$); the domain $D(K_m^B)$ is defined in [30], $K_m^B$ is Hermitian on $C_0^\infty (B)^2$ (proved to be essentially skew-selfadjoint in the case $d\ge 2$ in [30]).

Here, $G_m^B : H^{1/2}_m(B) \rightarrow H^{-1/2}_m(B)$ is the inverse Helmholtz operator on *B*, namely

94 $$\begin{aligned} G_m^B = E(-\nabla ^2 + m^2)^{-1}|_{H^{1/2}_m(B)}, \end{aligned}$$

with $E:H^{1/2}\rightarrow H^{1/2}_m(B)$ the orthogonal projection.

Then $K_m :D(K_m)\subset H_m\rightarrow H_m$ is defined as the closure of the complex linear extension of $K_m^B$ to $D(K_m) \equiv D(K_m^B)+ \imath _m D(K_m^B)$, and

$$\begin{aligned} A_m \equiv -\imath _m K_m \end{aligned}$$

is a Hermitian operator on $H_m$. Our aim is to show that

$$\begin{aligned} \log \Delta _{B,m} = -2\pi A_m,\quad m >0, \end{aligned}$$

also in the $d=1$ case. We note that $G^B_m$ is given by the following commutative diagram 
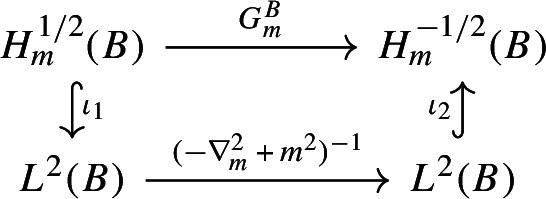
 where $\nabla ^2_m$ is the Laplacian on *B* with external boundary conditions in Appendix 6.3.

#### $m\ge 0$

We now set

$$\begin{aligned} H_0(B) \equiv \dot{H}_0(B)\oplus {{\mathbb {R}}}\subset {{\mathcal {H}}}_0 \equiv {\dot{{{\mathcal {H}}}}}_0 \oplus {{\mathbb {C}}}. \end{aligned}$$

$H_0(B)$ is a real Hilbert space with the direct sum scalar product. We choose a vector $u\in H_m(B)$, $u\notin \dot{H}_m(B)$. Clearly, the real linear identification $\dot{I} : \dot{H}_0(B) \rightarrow \dot{H}_m(B)$ extends to a real linear, topological identification $I : H_0(B) \rightarrow H_m(B)$ mapping $0\oplus 1$ to *u*. Namely *I* is a bounded, invertible real linear map $H_0(B) \rightarrow H_m(B)$. When we compare operators acting on $H_0(B)$ and on $H_m(B)$, we identify these two spaces and consider the operators acting on the same topological linear space $H_0(B) = H_m(B)$.

Let $\log \Delta _{B,m}$ and $\log {\dot{\Delta }}_{B,m}$ be the modular Hamiltonian of $H_m(B)\subset {{\mathcal {H}}}_m$ and of $\dot{H}_m(B)\subset {{\mathcal {H}}}_m$ respectively, $m >0$. In the massless case, let $\log {\dot{\Delta }}_{B,0}$ be the modular Hamiltonian of $\dot{H}_0(B)\subset {\dot{{{\mathcal {H}}}}}_0$ and set

$$\begin{aligned} \log \Delta _{B,0} \equiv \log {\dot{\Delta }}_{B,0}\oplus 0 \quad \mathrm{on}\ {{\mathcal {H}}}_0. \end{aligned}$$

Similarly, let $$D_m$$ be the polariser of $$H_m(B)$$, $$\dot{D}_m$$ the polariser of $$\dot{H}_m(B)$$, $$ m> 0$$. With $$\dot{D}_0$$ the polariser of $$\dot{H}_0(B)$$, set

$$\begin{aligned} D_0 \equiv \dot{D}_0 \oplus 0 \quad \mathrm{on}\ H_0(B). \end{aligned}$$

##### Lemma 5.10

95 $$\begin{aligned} \imath _m \tanh \big (\frac{1}{2}\log \Delta _{B,m}\big )|_{H^{1/2}_m(B)} - \imath _0 \tanh \big (\frac{1}{2}\log \Delta _{B,0}\big )|_{H^{1/2}_0(B)} \end{aligned}$$

is in $\mathcal{L}^1(H^{1/2}_m(B),H^{-1/2}_m(B))$. (With the identification $H_m(B) = H_0(B)$.)

##### Proof

By Proposition 5.8, $(\dot{D}^{-1}_m - \dot{D}^{-1}_0)|_{H^{-1/2}_m(B)}$ is in $\mathcal{L}^1$, so $\dot{D}_m - \dot{D}_0$ is in $\mathcal{L}^1$. By Lemma 3.8, $(D_m - D_0)|_{H^{1/2}_m(B)}$ is in $\mathcal{L}^1$ too. This is equivalent to requirement that the operator (95) is in $\mathcal{L}^1(H^{1/2}_m(B),H^{-1/2}_m(B))$. $\quad \square$

##### Lemma 5.11

The operator $\big ( -2\pi \imath _m A_m|_{H_m(B)} - \imath _0 \log \Delta _{B,0} |_{H_0(B)}\big )$ is in $\mathcal{L}^p$, $p>1$, $m>0$. Moreover, $K^B_m = \imath _m A_m|_{H_m(B)}$ is skew-selfadjoint on $H_m(B)$.

##### Proof

Since $\dot{H}_m(B)$ is closed and finite codimensional in $H_m(B)$, it suffices to show that

96 $$\begin{aligned} -2\pi \imath _m A_m|_{\dot{H}_m(B)} - \imath _0 \log {\dot{\Delta }}_{B,0} |_{\dot{H}_0(B)} \end{aligned}$$

is in $\mathcal{L}^p$, $p>1$. By (93) and (92), the operator (96) is equal to the sum of two operators

$$\begin{aligned} m^2\begin{bmatrix} 0 &{}\quad 0 \\ \frac{1}{2}(1 - x^2) &{}\quad 0 \end{bmatrix} + \frac{1}{2} m^2\begin{bmatrix} 0 &{}\quad 0 \\ G^B_m&{}\quad 0 \end{bmatrix} \end{aligned}$$

that are both in $\mathcal{L}^p$, $p>1$, see [30].

The skew-selfadjointness of $K^B_m$ then follows by [30, Prop. 2.1]. $\quad \square$

##### Theorem 5.12

The modular Hamiltonian $\log \Delta _{B,m}$ associated with the unit, time-zero interval *B* in the free scalar, massive, quantum field theory in $1+1$ dimension is given by

97 $$\begin{aligned} \imath _m\log \Delta _{B,m} = - 2\pi \left[ \begin{matrix} 0 &{}\quad \frac{1}{2}(1 - x^2) \\ \frac{1}{2}(1 - x^2)\big (\partial _x^2 -m^2\big ) - x \partial _x - \frac{1}{2} m^2 G^B_m&{}\quad 0 \end{matrix}\right] \end{aligned}$$

on $H_m(B)$, with $G_m^B : H^{1/2}_m(B) \rightarrow H^{-1/2}_m(B)$ the inverse Helmholtz operator on *B* (94).

##### Proof

By Lemma 5.11,

$$\begin{aligned} -2\pi \imath _m A_m|_{H_m(B)} - \imath _0 \log \Delta _{B,0} |_{H_0(B)} \end{aligned}$$

is in $\mathcal{L}^1$, thus

98 $$\begin{aligned} \imath _m\tanh (\pi A_m)|_{H_m(B)} - \imath _0 \tanh \big (\frac{1}{2} \log \Delta _{B,0}\big )|_{H_0(B)} \end{aligned}$$

is in $\mathcal{L}^p$, $p>1$, by Corollary 6.5, so it is compact.

By Lemma 5.10, also

99 $$\begin{aligned} \imath _m \tanh \big (\frac{1}{2}\log \Delta _{B,m}\big )|_{H^{1/2}_m(B)} - \imath _0 \tanh \big (\frac{1}{2} \log \Delta _{B,0}\big )|_{H^{1/2}_0(B)} \end{aligned}$$

is compact. Set

$$\begin{aligned} T \equiv \imath _m \tanh \big (\frac{1}{2}\log \Delta _{B,m}\big )|_{H_m(B)} - \imath _m\tanh (\pi A_m)|_{H_m(B)}; \end{aligned}$$

by (98) and (99), $T|_{H^{1/2}_m(B)}$ is compact. As $\Delta _{B,m}^{is}$ commutes with *T*, thus with $T^*T$, we infer that so $T|_{H^{1/2}_m(B)}$ is equal to zero because $\Delta _{B,m}$ has empty point spectrum [16]. This implies $-\imath _m 2\pi A_m|_{H^{1/2}_m(B)} = \imath _m \log \Delta _{B,m}|_{H^{1/2}_m(B)}$. As both these operators are skew-selfadjoint on $H_m(B)$, we have $-\imath _m 2\pi A_m|_{H^{1/2}_m(B)} = \imath _m \log \Delta _{B,m}|_{H^{1/2}_m(B)}$ on $H_m(B)$, thus on the intersection of $H_m(B) +\imath _m H_m(B)$ with the domain of $\log \Delta _{B,m}$ is a core for $\log \Delta _{B,m}$, being a dense $\Delta _{B,m}^{is}$-invariant subspace; and it is also a core for $A_m$ by the same argument. Thus

$$\begin{aligned} -\imath _m 2\pi A_m = \imath _m \log \Delta _{B,m}, \end{aligned}$$

namely (97) holds. $$\quad \square $$

### Local entropy of a Klein–Gordon wave packet, $d=1$

Although this section contains a main application of our paper, we shall be very short on its background as this is explained in details in [9, 30].

Let $\Phi$ be Klein–Gordon wave, $d=1$, $m>0$, with compactly supported, smooth Cauchy data *f*, *g*. Thus $\partial ^2_t\Phi -\partial ^2_x \Phi = - m^2\Phi$ and $f= \Phi |_{t=0}$, $g= \partial _t \Phi |_{t=0}$. The entropy $S_\Phi$ of $\Phi$ is given by

$$\begin{aligned} S_\Phi = \Im (\Phi , P_H i \log \Delta _H\, \Phi ). \end{aligned}$$

Here, $H = H_m(B)$, $\Delta _H$ is the modular operator and $P_H$ is the cutting projection associated with *H*. $\Phi$ is the vector $f\oplus g\in H_m = H_m^{1/2}\oplus H_m^{-1/2}$. Recall that the time-zero energy density of $\Phi$ is given by $\langle T^{(m)}_{00}\rangle _{\Phi } = \frac{1}{2} \big ( g^2 + (\partial _xf)^2 + m^2f^2 \big )$.

#### Theorem 5.13

The entropy $S_\Phi$ of the Klein–Gordon wave $\Phi$ in the unit interval $(-1,1)$ at time $t=0$ is given by

100 $$\begin{aligned} S_{\Phi } = 2\pi \int ^1_{-1} \frac{1 - x^2}{2} \langle T^{(m)}_{00}\rangle _{\Phi }\, dx +\pi m^2\int ^1_{-1}\int ^1_{-1} G_{m}(x - y)f(y) f(x)dx dy\nonumber \\ \end{aligned}$$

where $G_m$ is the Green function for the Helmholtz operator, $G_m(x) = \frac{1}{2m}e^{-m|x|}$.

#### Proof

The proof follows the one in the higher dimensional case; this is possible as we now have the formula for the local modular Hamiltonian. $\quad \square$

Note that the above results have a straightforward version with *B* replaced by any other interval, same as [30].

### Further consequences in QFT

In this section, we provide a few direct consequences in second quantisation of our results.

#### Local entropy of coherent states

By the analysis in [9, 29, 30], we have an immediate corollary in Quantum Field Theory concerning the local vacuum relative entropy of a coherent state.

Let ${{\mathcal {A}}}_m(B)$ be the von Neumann algebra associated with the unit space ball *B* (thus to the causal envelope *O* of *B*) by the free, neutral QFT on the Minkowski spacetime, $d\ge 1$, $m> 0$.

##### Corollary 5.14

Araki’s relative entropy $S(\varphi _\Phi |\!| \varphi )$ on ${{\mathcal {A}}}_m(O)$ (see [4]) between the vacuum state $\varphi$ and the coherent state $\varphi _\Phi$ associated with the one-particle wave $\Phi \in {{\mathcal {H}}}_m$ is given by (100).

##### Proof

The case $d\ge 2$ is proved in [30]. By applying Theorem 5.13, the corollary follows now in the $d=1$ case too as in [9, 29]. $\quad \square$

The formula for $S_\Phi$ is the same in the massless case, provided one deals with restricted Cauchy data as above, in order that $\Phi \in {{\mathcal {H}}}_0$, see [28, Sect. 4]. See also [10] for a discussion on relative entropy in a curved spacetime setting.

#### Type ${III}_1$ property

We show here the type ${III}_1$ factor property (see [41]) for the local von Neumann algebras associated with free, scalar QFT. In the massless case, this follows from [22]; in the massive case from [16], if $d>1$.

##### Proposition 5.15

${{\mathcal {A}}}_m(B)$ is a factor of type ${III}_1$, $d= 1$, $m >0$.

##### Proof

${{\mathcal {A}}}_m(B)$ is a factor because the symplectic form on $H_m(B)$ is non-degenerate. Concerning the type ${III}_1$ property, by [17] it suffices to show that the additive subgroup of ${{\mathbb {R}}}$ generated by $\mathrm{sp}_{e}(\log \Delta _{B,m})$ is equal to ${{\mathbb {R}}}$, with $\mathrm{sp}_{e}$ denoting the essential spectrum. Due to the relation (9), $\mathrm{sp}_{e}(\log \Delta _{B,m})$ is symmetric, so it is enough to show that $\mathrm{sp}_{e}(\tanh ^2(\frac{1}{2}\log \Delta _{B,m}))\supset {{\mathbb {R}}}_+$.

Now, $\tanh ^2(\frac{1}{2}\log \Delta _{B,m})$ is bounded, selfadjoint and leaves $H_m(B)$ invariant, so its essential spectrum is equal to $\mathrm{sp}_{e}\big (\tanh ^2(\frac{1}{2}\log \Delta _{B,m})|_{H_m(B)}\big )$ as real linear operator. By (16), we then have to show that $\mathrm{sp}_{e}(-D^2_m)\supset [0,1]$. Similarly as in Lemma 3.8, we have $\mathrm{sp}_{e}(D^2_m) = \mathrm{sp}_{e}(\dot{D}^2_m)$. On the other hand, $\mathrm{sp}_{e}(\dot{D}^2_m) = \mathrm{sp}_{e}(\dot{D}^2_0)$ because $\dot{D}^2_m - \dot{D}^2_0$ is compact by Thm. 5.12 and Thm. 6.3. We then conclude or proof by noticing that $\mathrm{sp}_{e}(-\dot{D}^2_0)\supset [0,1]$, because $\mathrm{sp}_{e}(\log \Delta _{B,0}) ={{\mathbb {R}}}$, see [27]. $\quad \square$

## Appendixes

### Functional calculus for real linear operators

The following proposition is part of Prop. 2.2 of [30]. Let ${{\mathcal {B}}}$ be the real algebra of complex, bounded Borel functions on ${{\mathbb {R}}}$ such that $f(-t) = {\bar{f}}(t)$

#### Proposition 6.1

Let ${{\mathcal {H}}}$ be a Hilbert space, $H\subset {{\mathcal {H}}}$ a closed, real linear subspace and $A :D(A)\subset {{\mathcal {H}}}\rightarrow {{\mathcal {H}}}$ a selfadjoint operator. With $K = iA$, the following are equivalent:

(i) $$e^{isA}H = H, \ s\in {\mathbb {R}}$$,
(ii) $$f(A)H \subset H$$, $$f\in {{\mathcal {B}}}$$,
(iii) $D(K)\cap H$ is dense in *H*, $K(D(K)\cap H)\subset H$ and $K: (D(K)\cap H) \subset H\rightarrow H$ is skew-selfadjoint on *H*.

If *A* and *H* are as in Proposition 6.1, we shall say that *H* is *iA*-invariant.

Let now *H* be a real Hilbert space and $H_{{\mathbb {C}}}$ the complexified Hilbert space, namely $H_{{\mathbb {C}}}= H\oplus H$ with complex structure $$\iota = \begin{bmatrix} 0 &{}\quad -1\\ 1 &{}\quad 0 \end{bmatrix}$$. We write elements $x\in H_{{\mathbb {C}}}$ as $x = \xi + \iota \eta$, $\xi , \eta \in H$. We have

$$\begin{aligned}&(\xi + \iota \eta , \xi ' + \iota \eta ') = (\xi , \xi ') + (\eta ,\eta ') + i(\xi , \eta ') - i (\eta , \xi ' ),\\&||\xi + \iota \eta ||^2 = ||\xi ||^2 + ||\eta ||^2. \end{aligned}$$

Let *T* be a real linear, bounded operator on *H*. We denote by $\check{T}$ its promotion to $H_{{\mathbb {C}}}$:

$$\begin{aligned} \check{T} : \xi + \iota \eta \mapsto T\xi + \iota T\eta , \end{aligned}$$

namely $\check{T}$ is the unique complex linear operator on $H_{{\mathbb {C}}}$ that restricts to *T* on *H*. Then $||\check{T} || = || T||$ because

$$\begin{aligned} ||\check{T}(\xi + \iota \eta )||^2 = ||T\xi ||^2 + ||T\eta ||^2 \le ||T||^2(||\xi ||^2 + ||\eta ||^2) = ||T||^2\, ||\xi + \iota \eta ||^2 . \end{aligned}$$

Note that

$$\begin{aligned} T\in \mathcal{L}^2(H) \Leftrightarrow \check{T}\in \mathcal{L}^2(H_{{\mathbb {C}}}), \end{aligned}$$

indeed $||\check{T}||^2_2 = ||T||_2^2$ because a real orthonormal basis $\{e_k\}$ for *H* is also a complex orthonormal basis for $H_{{\mathbb {C}}}$ and

$$\begin{aligned} ||\check{T}||^2_2 = ||T||_2^2 = \sum _k ||Te_k||^2. \end{aligned}$$

Assume that *T* is skew-selfadjoint on *H*, namely $T^* = -T$. Then $\check{T}$ is skew-selfadjoint as complex linear operator on $H_{{\mathbb {C}}}$, so $\iota \check{T}$ is a bounded selfadjoint operator on $H_{{\mathbb {C}}}$. With *f* a continuous complex function on ${{\mathbb {R}}}$, we may define the complex linear operator $f(\iota \check{T})$ on $H_{{\mathbb {C}}}$ by the usual continuous functional calculus. Let then $f\in {{\mathcal {B}}}$; by Proposition 6.1 we have

$$\begin{aligned} f(\iota \check{T}) H \subset H. \end{aligned}$$

#### Proposition 6.2

Let $H\subset {{\mathcal {H}}}$ be a standard subspace and *T* a skew selfadjoint operator on *H* as above. Suppose that

101 $$\begin{aligned} T = i X |_H \end{aligned}$$

with *X* a selfadjoint operator on ${{\mathcal {H}}}$. With $A= -\iota \check{T}$ the selfadjoint operator on $H_{{\mathbb {C}}}$ as above, we have

102 $$\begin{aligned} f(A)|_H = f(X) |_H, \end{aligned}$$

for every $$f\in {{\mathcal {B}}}$$.

#### Proof

The statement holds if $f(x) = e^{ix}$ because *T* is the infinitesimal skew-selfadjoint generator of $e^{isA}|_H = e^{isX}|_H$. So it holds if *f* is the Fourier transform of a real $L^1$-function *g* as

$$\begin{aligned} f(A)|_H = \int g(s) e^{-isA}|_H ds = \int g(s) e^{-isX}|_H ds = f(X)|_H \end{aligned}$$

Then (102) holds for every continuous function with compact support $f\in {{\mathcal {B}}}$, as it can be uniformly approximated by functions as above by the Stone-Weierstrass theorem.

Let now *f* be any function in ${{\mathcal {B}}}$ and fix two vectors $\xi ,\eta \in H$. There exists a uniformly bounded sequence of continuous functions $f_n\in {{\mathcal {B}}}$ with compact support such that $f_n\rightarrow f$ almost everywhere with respect to the spectral measures of *A* and *X* associated with $\xi ,\eta$. Then

$$\begin{aligned} (\xi , f(A)\eta ) = \lim _n (\xi , f_n(A)\eta ) = \lim _n (\xi , f_n(X)\eta ) = (\xi , f(X)\eta ) \end{aligned}$$

by the Lebesgue dominated convergence theorem, that concludes our proof because $\xi ,\eta$ are arbitrary.

### Operator Lipschitz perturbations

The next theorem is due to Potatov and Sukochev [35].

#### Theorem 6.3

Let $A_1, A_2$ be selfadjoint operators on a Hilbert space ${{\mathcal {H}}}$ and *f* a uniformly Lipschitz function on ${{\mathbb {R}}}$. If $A_1 - A_2\in \mathcal{L}^p({{\mathcal {H}}})$, with $p >1$, then also $f(A_1) - f(A_2)\in \mathcal{L}^p({{\mathcal {H}}})$.

Note that, in Thm. 6.3, it suffices to assume that $(A_1 - A_2)|_{{\mathcal {D}}}\in \mathcal{L}^p({{\mathcal {H}}})$ with ${{\mathcal {D}}}$ a core for $A_1$ or $A_2$, since then ${{\mathcal {D}}}$ is a core for both $A_1$ or $A_2$ and $D(A_1) = D(A_2)$ because $A_1 - A_2$ is bounded.

The following corollary was communicated to us by F. Sukochev.

#### Corollary 6.4

Let $A_k$ be a selfadjoint operator on the Hilbert space ${{\mathcal {H}}}_k$, $k=1,2$, and suppose that ${{\mathcal {H}}}_1$ and ${{\mathcal {H}}}_2$ are the same topological vector space, that we call ${{\mathcal {H}}}$. Then

$$\begin{aligned} A_1 - A_2\in \mathcal{L}^p({{\mathcal {H}}})\implies f(A_1) - f(A_2)\in \mathcal{L}^p({{\mathcal {H}}}), \end{aligned}$$

$p>1$, for every uniformly Lipschitz function *f* on ${{\mathbb {R}}}$.

#### Proof

Let $C: {{\mathcal {H}}}_1 \rightarrow {{\mathcal {H}}}_2$ be the complex linear identification of ${{\mathcal {H}}}_1$ and ${{\mathcal {H}}}_2$ as topological vector spaces. So *C* is a bounded operator with bounded inverse $C^{-1}$. Then we have to show that

$$\begin{aligned} A_1 - C^{-1} A_2 C\in \mathcal{L}^p({{\mathcal {H}}}_1) \implies f(A_1) - C^{-1} f(A_2) C\in \mathcal{L}^p({{\mathcal {H}}}_1), \end{aligned}$$

or, equivalently, that

$$\begin{aligned} CA_1 - A_2 C\in \mathcal{L}^p({{\mathcal {H}}}_1,{{\mathcal {H}}}_2) \implies C f(A_1) - f(A_2) C\in \mathcal{L}^p({{\mathcal {H}}}_1,{{\mathcal {H}}}_2). \end{aligned}$$

With ${{\mathcal {K}}}= {{\mathcal {H}}}_1\oplus {{\mathcal {H}}}_2$, the operator $A = A_1\oplus A_2$ is selfadjoint on ${{\mathcal {K}}}$. Set $$V = \begin{bmatrix} 0 &{}\quad 0\\ C &{}\quad 0\end{bmatrix}$$; then

$$\begin{aligned} V A - A V= \begin{bmatrix} 0 &{}\quad 0\\ CA_1 - A_2 C &{}\quad 0\end{bmatrix} \end{aligned}$$

and

$$\begin{aligned} V f(A) - f(A) V = \begin{bmatrix} 0 &{}\quad 0\\ Cf(A_1) - f(A_2) C &{}\quad 0\end{bmatrix}, \end{aligned}$$

so we have to show that

$$\begin{aligned} V A - A V\in \mathcal{L}^p({{\mathcal {K}}})\implies V f(A) - f(A) V\in \mathcal{L}^p({{\mathcal {K}}}), \end{aligned}$$

that follows by [35, Eq. (14)]. $\quad \square$

We need a certain real version of Corollary 6.4.

#### Corollary 6.5

Let $H_k\subset {{\mathcal {H}}}_k$ be a standard subspace and $X_k$ a selfadjoint operator on ${{\mathcal {H}}}_k$ such that $H_k$ is $i_k X_k$-invariant, $k =1,2$. Suppose that $H_1$ and $H_2$ are the same real linear space *H* with equivalent scalar products. Then

$$\begin{aligned} i_1 X_1|_H - i_2 X_2|_H \in \mathcal{L}^p(H) \implies i_1 f(X_1)|_H - i_2 f(X_2)|_H \in \mathcal{L}^p(H) , \end{aligned}$$

$p>1$, for every uniformly Lipschitz function *f* on ${{\mathbb {R}}}$ such that $f(-x) = -\overline{f(x)}$.

#### Proof

Let ${H_k}_{{\mathbb {C}}}$ be the usual complexification of the real Hilbert space $H_k$. Then ${H_1}_{{\mathbb {C}}}$ and ${H_2}_{{\mathbb {C}}}$ are equivalent complex Hilbert spaces.

Let $A_k$ be the selfadjoint extension of $X_k$ to ${H_k}_{{\mathbb {C}}}$ as above; by Proposition 6.2, we have

$$\begin{aligned}&i_1 X_1|_H - i_2 X_2|_H\in \mathcal{L}^p(H)\implies A_1 - A_2 \in \mathcal{L}^p(H_{{\mathbb {C}}})\\&\quad \implies \iota f(A_1) - \iota f(A_2) \in \mathcal{L}^p(H_{{\mathbb {C}}})\\&\quad \implies \iota f(A_1)|_H - \iota f(A_2)|_H \in \mathcal{L}^p(H)\implies i_1 f(X_1)|_H - i_2 f(X_2)|_H \in \mathcal{L}^p(H) . \end{aligned}$$

$$\quad \square $$

### Extensions of the Laplacian via Helmholtz operator

Let ${{\mathcal {H}}}$ be a Hilbert space, ${{\mathcal {K}}}$ a closed subspace and $A: D(A)\subset {{\mathcal {H}}}\rightarrow {{\mathcal {H}}}$ a positive selfadjoint linear operator.

$$\begin{aligned} D_0 = \big \{\xi \in D(A)\cap {{\mathcal {K}}}: A\xi \in {{\mathcal {K}}}\big \} \end{aligned}$$

is dense in ${{\mathcal {K}}}$ and denote by $A_0$ the restriction of *A* to $D_0$, as operator ${{\mathcal {K}}}\rightarrow {{\mathcal {K}}}$. Clearly $A_0$ is a positive Hermitian operator on ${{\mathcal {K}}}$. We want to study the selfadjoint extensions of $A_0$.

Choose $m>0$, then $(A + m^2)^{-1}$ is a bounded selfadjoint operator on ${{\mathcal {H}}}$ whose norm is $||(A + m^2)^{-1}||\le 1/m^2$. With *E* the orthogonal projection of ${{\mathcal {H}}}$ onto ${{\mathcal {K}}}$, set

103 $$\begin{aligned} T = E(A +m^2)^{-1}|_{{\mathcal {K}}}. \end{aligned}$$

Then *T* is a bounded, selfadjoint operator on ${{\mathcal {K}}}$ and $||T||\le 1/m^2$. We have

104 $$\begin{aligned} T(A_0 + m^2) \xi = \xi ,\quad \xi \in D_0. \end{aligned}$$

We note the following.

- $\ker (T) = \{0\}$. Let $\xi \in {{\mathcal {K}}}$; since $T\xi = 0$ implies
  $$\begin{aligned} (\xi , T\xi )= & {} (\xi , E(A + m^2)^{-1}\xi ) = (\xi , (A +m^2)^{-1}\xi ) \\= & {} ((A +m^2)^{-1/2}\xi , (A +m^2)^{-1/2}\xi )=0, \end{aligned}$$
  we have
  $$\begin{aligned} T\xi = 0 \implies (A +m^2)^{-1/2}\xi = 0 \implies \xi = 0. \end{aligned}$$
- Let $A_m$ be defined by $(A_m + m^2) \equiv T^{-1}$. Then $A_m$ is a positive, selfadjoint extension of $A_0$ on ${{\mathcal {K}}}$ and $A_m \ge m^2$. Indeed, Eq. (104) implies
  $$\begin{aligned} T^{-1}\xi = (A_0 + m^2)\xi , \quad \xi \in D_0. \end{aligned}$$
- By theorems of von Neumann, Krein, Friedrichs et al. (see [1, 2, 38]), every positive selfadjoint extension of $A_0$ lies between $A_{\min }$ and $A_{\max }$, where where $A_{\min }$ and $A_{\max }$ are respectively the Krein and the Friedrichs extension of $A_0$ on ${{\mathcal {K}}}$. In particular,
  105 $$\begin{aligned} A_{\min } \le A_m \le A_{\max }, \end{aligned}$$
  in the quadratic form sense.

Consider now the case of ${{\mathcal {K}}}= L^2(B)\subset {{\mathcal {H}}}= L^2({{\mathbb {R}}}^d)$. If $f\in {{\mathcal {C}}}^\infty (\partial B)$, consider the exterior Dirichlet problem for the Helmholtz operator: find a smooth function $f^c$ on the complement $B^c$ of *B* such that:

$$\begin{aligned} f^c |_{\partial B} = f, \quad (\nabla ^2 - m^2)f^c = 0 \ \text {on the complement of}\ {\bar{B}}; \end{aligned}$$

this problem is studied e.g. [33].

Denote by $C_m$ the space of all $f\in C^\infty (\partial B)$ such that $f^c$ exists with $f^c$ and partial derivatives of all order tending to zero as $r = |x| \rightarrow +\infty$ faster than any inverse power of *r*. In this case the solution $f^c$ is unique by the maximum principle.

For completeness, we sketch the following proposition, although it is not needed in this form in the paper (we need Corollary 6.7).

#### Proposition 6.6

Let ${{\mathcal {H}}}= L^2({{\mathbb {R}}}^d)$, ${{\mathcal {K}}}= L^2(B)$, and $A = -\nabla ^2$ be the Laplacian on $L^2({{\mathbb {R}}}^d)$; then

$$\begin{aligned} A_m = -\nabla ^2_m, \end{aligned}$$

where $\nabla ^2_m$ is the Laplacian on $L^2(B)$ with boundary condition

$$\begin{aligned} \partial ^-_r f = - \partial ^+_r f^c\ \mathrm{on}\ \partial B, \end{aligned}$$

more precisely, $D_m \equiv \big \{f\in C^\infty ({\bar{B}}) : f|_{\partial B}\in C_m,\ \partial ^-_r f = -\partial ^+_r f^c\ \mathrm{on}\ \partial B\big \}$ is a core for $A_m$, with $\partial _r^\pm$ denoting the outer/inner normal derivative.

#### Proof

Let $g\in C_0^\infty (B)$ and $f = (A +m^2)^{-1}g$. Then $f\in D(\nabla ^2)$ and *f* is a solution of the equation $(-\nabla ^2 + m^2)f = g$ on ${{\mathbb {R}}}^d$. In particular $(-\nabla ^2 + m^2)f = 0$ on $B^c$, namely $f|_{B^c} = {(f |_{\partial B}})^c$. As $g\in C_0^\infty (B)$, *f* belongs to the Schwarz space $S({{\mathbb {R}}}^d)$, thus $f|_{B^c}\in C_m$.

With *T* given by (103), we have $Tg = f|_{{\bar{B}}}$; as *T* is a bounded operator on $L^2(B)$ and $C_0^\infty (B)$ is dense in $L^2(B)$, the domain $T C_0^\infty (B)$ is a core for $A_m = T^{-1}$. Since $T C_0^\infty (B)\subset D_m$, we have that $A_m$ is essentially selfadjoint on $D_m$. Clearly, $A_m = -\nabla _m^2$ on $D_m$.

Now $-\nabla _m^2$ is Hermitian on $D_m$ by the Green identity (consider the integration on the boundary of a corona $1\le r\le R$ and then let $R\rightarrow \infty$), so we conclude that $A_m = -\nabla _m^2$ because selfdajoint operators are maximal Hermitian. $\quad \square$

The requirement $f^c\in L^2(B^c)$ in the definition of $D_m$ is probably automatic. Let us be more explicit in the $d=1$ case. In this case, $B = (-1,1)$. If *f* is a smooth solution of $(-\nabla ^2 + m^2) f = 0$, with $\nabla = \frac{d}{dx}$ in $[1,\infty )$, then $f(x) = C_+ e^{m x} + C_- e^{-m x}$, with $C_\pm$ constant. Thus $f(x) = C_- e^{-m x}$ if $f\in L^2(1,\infty )$. Similarly, $f(x) = C_+ e^{m x}$ in the $(-\infty , -1]$ case. Therefore $\nabla ^\mp f(\pm 1) = m f(\pm 1)$ and

$$\begin{aligned} D_m \equiv \big \{f\in C^\infty ([-1,1]): \nabla ^\mp f(\pm 1) = m f(\pm 1)\big \}. \end{aligned}$$

#### Corollary 6.7

$E(\nabla ^2 - m^2)^{-1}|_{L^2(B)}\in \mathcal{L}^p(L^2(B))$ iff $p > d/2$, with *E* the orthogonal projection onto $L^2(B)$.

#### Proof

Let $A_0= -\nabla ^2 + m^2$ on $C^\infty _0(B)$; then $A_{\min }=-\nabla _D^2 + m^2$ and $A_{\max }=-\nabla _K^2 + m^2$, where $\nabla _D^2$ and $\nabla _K^2$ are the Dirichlet and the Krein Laplacian. Now $\nabla _D^2$ satisfies the Weyl asymptotic, so $(\nabla _D^2 - m^2)^{-1}\in \mathcal{L}^p$ iff $p > d/2$, see [13]. Moreover, the same asymptotic hold for $(\nabla _K^2 - m^2)^{-1}$, see [19]. By the min-max principle (see [38, Sect. 12.1]), the same asymptotic holds for every positive, selfadjoint extension of the Laplacian on $C_0^\infty (B)$, in particular for $\nabla _m = E(\nabla ^2 - m^2)^{-1}|_{L^2(B)}$, so our statement holds. $\quad \square$
